# Skilful nowcasting of extreme precipitation with NowcastNet

**DOI:** 10.1038/s41586-023-06184-4

**Published:** 2023-07-05

**Authors:** Yuchen Zhang, Mingsheng Long, Kaiyuan Chen, Lanxiang Xing, Ronghua Jin, Michael I. Jordan, Jianmin Wang

**Affiliations:** 1 School of Software, BNRist, Tsinghua University, Beijing, China; 2 China Meteorological Administration, Beijing, China; 3 University of California, Berkeley, CA USA

**Keywords:** Computer science, Computational science, Atmospheric science

## Abstract

Extreme precipitation is a considerable contributor to meteorological disasters and there is a great need to mitigate its socioeconomic effects through skilful nowcasting that has high resolution, long lead times and local details^1–3^. Current methods are subject to blur, dissipation, intensity or location errors, with physics-based numerical methods struggling to capture pivotal chaotic dynamics such as convective initiation^4^ and data-driven learning methods failing to obey intrinsic physical laws such as advective conservation^5^. We present NowcastNet, a nonlinear nowcasting model for extreme precipitation that unifies physical-evolution schemes and conditional-learning methods into a neural-network framework with end-to-end forecast error optimization. On the basis of radar observations from the USA and China, our model produces physically plausible precipitation nowcasts with sharp multiscale patterns over regions of 2,048 km × 2,048 km and with lead times of up to 3 h. In a systematic evaluation by 62 professional meteorologists from across China, our model ranks first in 71% of cases against the leading methods. NowcastNet provides skilful forecasts at light-to-heavy rain rates, particularly for extreme-precipitation events accompanied by advective or convective processes that were previously considered intractable.

## Abstract (web summary)

A new nowcasting model unifies physical-evolution schemes and deep-learning methods to accurately predict precipitation with lead times of up to 3 h, including extreme-precipitation events and weather systems that were previously considered intractable with physics-based numerical methods.

## Main

Nowcasting is defined by the World Meteorological Organization (WMO) as forecasting that yields local details across the mesoscale and small scale, over a period from the present up to 6 h ahead and which provides a detailed description of the present weather^1^. Nowcasting is crucial in risk prevention and crisis management of extreme precipitation, commonly defined as the 95th percentile of the cumulative frequency distribution of daily precipitation^2^. According to a recent report from the WMO^3^, over the past 50 years, more than 34% of all recorded disasters, 22% of related deaths (1.01 million) and 57% of related economic losses (US$ 2.84 trillion) were consequences of extreme-precipitation events.

Weather radar echoes provide cloud observations at sub-2-km spatial resolution and up to 5-min temporal resolution, which are ideal for precipitation nowcasting^6^. The natural option for exploiting these data is numerical weather prediction, which produces precipitation forecasts based on solving coupled primitive equations of the atmosphere^7^. However, these methods, even when implemented on a supercomputing platform, restrict the numerical weather prediction forecast update cycles to hours and the spatial resolution to the mesoscale, whereas extreme weather processes typically exhibit lifetimes of tens of minutes and individual features at the convective scale^4,8,9^. Alternative methods such as DARTS^10^ and pySTEPS^9^ are based on an advection scheme inspired solely by the continuity equation. These methods solve separately for the future states of the motion fields and the intensity residuals from composite radar observations and iteratively advect past radar fields to predict future fields. The advection scheme partially respects the physical conservation laws of precipitation evolution and is able to provide skilful extrapolations within 1 h, but it degrades quickly beyond that horizon, incurring high location error and losing small convective features. These errors accumulate in the autoregressive advection processes in uncontrolled ways^11^, owing to existing advection implementations failing to incorporate nonlinear evolution simulations and end-to-end forecast error optimization.

Deep-learning methods have been applied in recent years to weather nowcasting^12–16^. These methods exploit large corpora of composite radar observations to train neural-network models in an end-to-end fashion, dispensing with explicit reference to the physical laws behind precipitation processes. They have proved useful for low-intensity rainfall as measured by per-grid-cell metrics such as the Critical Success Index (CSI)^4^. A large step forward in this setting has been the deep generative model of radar (DGMR) approach developed by DeepMind and the UK Met Office^4^. This approach generates spatiotemporally consistent predictions with a lead time of up to 90 min, simultaneously capturing chaotic convective details and accounting for ensemble forecast uncertainty. In an expert evaluation by more than 50 meteorologists from the UK Met Office, DGMR ranked first in 89% of cases against competing methods, including the advection-based method pySTEPS^9^. Still, for extreme precipitation, DGMR may produce nowcasts with unnatural motion and intensity, high location error and large cloud dissipation at increasing lead times^4^. These problems reflect the fact that radar echoes are only partial observations of the atmospheric system. Deep-learning models based purely on radar data analysis are hampered in their ability to capture the fuller range of physical phenomena underlying precipitation^5^. We believe that physical knowledge of aspects of precipitation processes, including the conservation law of cloud transport^10^ and the log-normal distribution of rain rate^17^, need to be embedded into data-driven models to make skilful nowcasting of extreme precipitation possible.

We present NowcastNet, a unified nowcasting model for extreme precipitation based on composite radar observations. It combines deep-learning methods with physical first principles, by means of a neural-network framework that implements neural evolution operators for modelling nonlinear processes and a physics-conditional mechanism for minimizing forecast error. This framework enables seamless integration of advective conservation into a learning model, successfully predicting long-lived mesoscale patterns and capturing short-lived convective details with lead times of up to 3 h. As we will show on the USA and China events corpora, the forecasts made by NowcastNet are judged by expert meteorologists to be more accurate and instructive than pySTEPS, DGMR or other deep-learning systems.

## NowcastNet

**Fig. 1:**
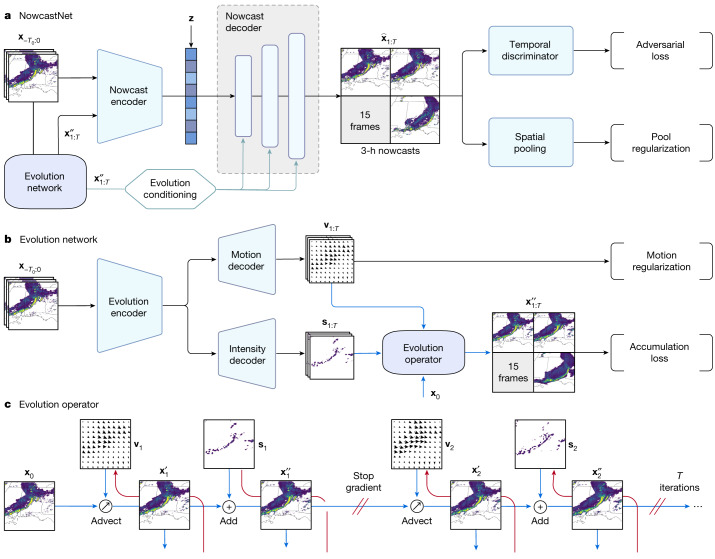
NowcastNet for extreme-precipitation nowcasting. **a**, Architecture of NowcastNet, a physics-conditional deep generative model. The nowcast encoder learns contextual representations. The nowcast decoder conditions on the physics-informed evolutions ${{\bf{x}}}_{1:T}^{{\prime\prime} }$ and transforms draws from a latent Gaussian vector **z** into mesoscale and convective-scale predictions ${\hat{{\bf{x}}}}_{1:T}$. **b**, Evolution network, a neural implementation of the advection schemes informed by the 2D continuity equation, which imposes compliance with the precipitation physics and outputs mesoscale predictions ${{\bf{x}}}_{1:T}^{{\prime\prime} }$. **c**, Evolution operator, a neural operator that iteratively advects **x**_0_ by motion fields **v**_1:*T*_ to get ${{\bf{x}}}_{1:T}^{{\prime} }$ and adds by intensity residuals **s**_1:*T*_ to get ${{\bf{x}}}_{1:T}^{{\prime\prime} }$. Precipitation data obtained from the MRMS^26^ dataset and maps produced with cartopy and Natural Earth.

Skilful nowcasting requires making use of both physical first principles and statistical-learning methods. NowcastNet provides such a unification using a neural-network framework, allowing end-to-end forecast error optimization. Our nowcasting algorithm (Fig. 1a) is a physics-conditional deep generative model that exploits radar-based estimates of surface precipitation to predict future radar fields ${\hat{{\bf{x}}}}_{1:T}$ given past radar fields ${{\bf{x}}}_{-{T}_{0}:0}$. The model includes a stochastic generative network parameterized by *θ* and a deterministic evolution network parameterized by *ϕ*. The nowcasting procedure is based on physics-conditional generation from latent random vectors **z**, described by

1 $$P({\hat{{\bf{x}}}}_{1:T}|{{\bf{x}}}_{-{T}_{0}:0},\phi \,;\theta )=\int \,P({\hat{{\bf{x}}}}_{1:T}|{{\bf{x}}}_{-{T}_{0}:0},\phi ({{\bf{x}}}_{-{T}_{0}:0}),{\bf{z}}\,;\theta )P({\bf{z}}){\rm{d}}{\bf{z}}.$$

The integration over latent Gaussian vectors **z** enables ensemble forecast with predictions skilfully capturing the pivotal chaotic dynamics^4^.

Although our work fits in a nascent thread of research on physics-informed neural networks^5^, there are many challenges in the precipitation domain that are not readily accommodated by existing research. Most notably, the multiscale nature of atmospheric physics introduces emergent dependencies among several spatiotemporal scales and imposes inherent limits on atmospheric predictability^8^. In particular, the convective processes are subject to chaotic error growth from uncertain initial conditions, limiting advection schemes to a spatial scale of 20 km and a lead time of 1 h (ref. ^18^). Naive combinations of neural networks and physical principles entangle the multiscale variability and corrupt the mesoscale and convective-scale patterns, creating undesirable confounding and uncontrolled errors.

We address the multiscale problem by a new conditioning mechanism that the data-driven generative network *θ* boosts over the advection-based evolution network *ϕ* (Fig. 1a). The evolution network imposes compliance with the physics of precipitation, yielding physically plausible predictions ${{\bf{x}}}_{1:T}^{{\prime\prime} }$ for advective features at a scale of 20 km. The nowcast decoder takes the nowcast encoder representations of past radar fields ${{\bf{x}}}_{-{T}_{0}:0}$, along with the evolution network predictions ${{\bf{x}}}_{1:T}^{{\prime\prime} }$, and generates fine-grained predictions ${\hat{{\bf{x}}}}_{1:T}$ from latent Gaussian vectors **z** that can capture convective features at a 1–2-km scale. Such a scale disentanglement mitigates error propagating upscale or downscale in the multiscale prediction framework^19^. We use the spatially adaptive normalization technique^20^ to enable an adaptive evolution conditioning mechanism. In each forward pass, the mean and variance of every-decoder-layer activations are replaced by the spatially corresponding statistics computed from the evolution network predictions ${{\bf{x}}}_{1:T}^{{\prime\prime} }$. As a result, NowcastNet adaptively combines mesoscale patterns governed by physical laws and convective-scale details revealed by radar observations, yielding skilful multiscale predictions with up to a 3-h lead time.

Learning is framed as the training of a conditional generative adversarial network^21^, given the pre-trained evolution network that encodes physical knowledge. A temporal discriminator is built on the nowcast decoder, taking as input the pyramid of features in several time windows and outputting whether the input is likely to be real radar or a fake field. The nowcast encoder and decoder are trained with an adversarial loss to generate convective details present in the radar observations but left out by the advection-based evolution network. Also, the generated nowcasts need to be spatially consistent with the radar observations. This is achieved by the pool regularization, which enforces consistency between spatial-pooled ensemble nowcasts and spatial-pooled observations. The pooling-level consistency is more tolerant of the spatial chaos in real fields and is capable of resolving the conflict between the generative network and the evolution network.

## Evolution network

NowcastNet enables multiscale nowcasting by conditioning the data-driven (stochastic) generative network *θ* on the advection-based (deterministic) evolution network *ϕ*. In atmospheric physics, the continuity equation is the fundamental conservation law governing the cloud transport and precipitation evolution. It has inspired a series of operational advection schemes^22^, which model the precipitation evolution as a composition of advection by motion fields and addition by intensity residuals. However, previous implementations of advection schemes, for example, pySTEPS, fall short in three respects: (1) their advection operation is not differentiable and thus cannot be embedded easily into an end-to-end neural framework for gradient-based optimization; (2) their steady-state assumption limits the implementations to linear regimes, failing to provide the nonlinear modelling capability crucial for precipitation simulations; and (3) their autoregressive nature prevents direct optimization of the forecast errors and errors arising from the estimation of the initial states, motion fields and intensity residuals will accumulate in an uncontrolled manner in the Lagrangian persistence model^8^.

We address these desiderata with our evolution network (Fig. 1b), which implements the 2D continuity equation^10^ through neural evolution schemes. On the basis of a new differentiable neural evolution operator, it learns the motion fields, intensity residuals and precipitation fields simultaneously by neural networks; moreover, it directly optimizes the forecast error throughout the time horizon by gradient-based backpropagation.

Our physics-informed evolution network is built on a new differentiable neural evolution operator (Fig. 1c). The evolution operator takes the current radar field **x**_0_ as input and predicts the future radar fields **x**_1:*T*_. At each time step, the radar field predicted at the last time step ${{\bf{x}}}_{t-1}^{{\prime\prime} }$ is evolved by one step of advection with the motion field **v**_*t*_ to obtain ${{\bf{x}}}_{t}^{{\prime} }$ and the intensity residual **s**_*t*_ is then added to yield ${{\bf{x}}}_{t}^{{\prime\prime} }$. The operator makes all motion fields and intensity residuals learnable end to end by gradient-based optimization, which is unattainable by existing advection schemes. When learning the operator with backpropagation, we stop the gradients between each time step to block information interference. This mitigates the numerical instability arising from the underdetermined nature of the overall system, which has discontinuous interpolations in the evolution operator.

The evolution network augments with an encoder–decoder architecture that simultaneously predicts motion fields **v**_1:*T*_ and intensity residuals **s**_1:*T*_ at all future time steps based on past radar fields ${{\bf{x}}}_{-{T}_{0}:0}$. Such a full dependency between the past and future time steps mitigates the nonstationarity issue in sequence prediction. Also, the evolution encoder, motion decoder and intensity decoder are neural networks (Fig. 1b), enabling nonlinear evolution modelling, which previous advection schemes struggle to capture.

Learning of the evolution network is framed as directly optimizing the forecast error throughout the time horizon. The accumulated error arises in the evolution operator, measured by the sum of distances between evolved field ${{\bf{x}}}_{t}^{{\prime\prime} }$ and the observed radar **x**_*t*_. Because each evolution step involves solving for both the motion field **v**_*t*_ and the intensity residual **s**_*t*_, to shortcut the gradient path for end-to-end optimization, we adopt the concept of residual learning^23^ and further calculate the sum of distances between the advected field ${{\bf{x}}}_{t}^{{\prime} }$ and the observed radar **x**_*t*_. Combining the two sums of distances leads to the accumulation loss. Furthermore, inspired in part by the continuity equation and in part by the fact that large precipitation patterns tend to be longer lived than small ones^8^, we further design a motion-regularization term to make the motion fields smoother on the grids with heavier precipitation. Specifically, the spatial gradients of the motion fields **v**_1:*T*_ are computed by a Sobel filter^24^ and the gradient norm, weighted by rain rate, is used as the regularizer.

## Evaluation settings

We evaluate the forecasting skill and value of NowcastNet against state-of-the-art precipitation nowcasting models. pySTEPS^9^, an advection-based method, has been widely adopted by meteorological centres worldwide for operational nowcasting^25^. PredRNN^13^, a data-driven neural network, has been deployed at the China Meteorological Administration. DGMR^4^, an ensemble nowcasting method based on deep generative models with integrated domain knowledge, for example, spatiotemporal consistency of clouds and heavy-tailed distribution of rainfall, has shown the best forecasting skill and value in an expert evaluation held by the UK Met Office.

All models are trained and tested on large radar corpora of the USA and China events, consisting of crops in fixed-length series extracted from the radar stream. An importance-sampling strategy^4^ is used to create datasets more representative of extreme-precipitation events. In the USA corpus, we use the Multi-Radar Multi-Sensor (MRMS) dataset^26^ and all models are trained with radar observations for the years 2016–2020 and evaluated for the year 2021. In the China corpus, we use a private dataset provided by the China Meteorological Administration, with radar observations from September 2019 to March 2021 for training and from April 2021 to June 2021 for evaluation. Although the China corpus is smaller, the underlying weather system is more complex owing to geographical diversity. To avoid overfitting, we use a transfer learning strategy^27^, in which all models are pre-trained on the USA training set and fine-tuned to the China training set.

NowcastNet can produce high-resolution fields in seconds at inference time. We report two main quantitative metrics: the CSI with neighbourhood^28^ that measures the location accuracy of nowcasts and the power spectral density (PSD)^29^ that measures the precipitation variability based on spectral characteristics of nowcasts compared with that of radar observations.

## Precipitation events

**Fig. 2:**
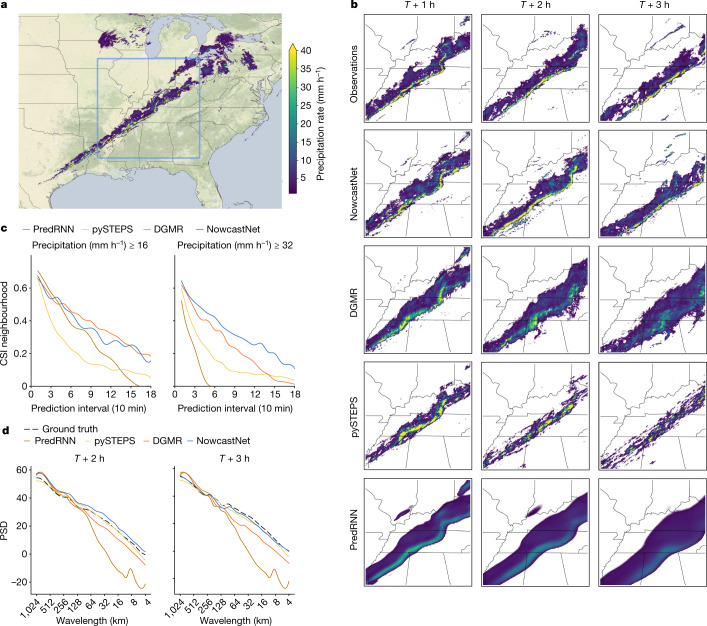
Case study of a precipitation event starting on 11 December 2021, with a large convective fine line and a tornado outbreak in eastern USA. NowcastNet is able to predict the convective-fine-line evolutions and details for a longer time period. **a**, Geographic context for the predictions. **b**, A single prediction at *T* + 1 h, *T* + 2 h and *T* + 3 h lead times for different models. **c**, CSI neighbourhood at thresholds 16 mm h^−1^ and 32 mm h^−1^. **d**, PSD at different wavelengths. Images are zoomed in 768 km × 768 km to highlight local details. Precipitation data obtained from the MRMS^26^ dataset and maps produced with cartopy and Natural Earth. Source data

We investigate a precipitation event starting at 09:30 UTC on 11 December 2021 (Fig. 2), which was part of a tornado outbreak in eastern USA. First, several lines of intense storm developed across the Mississippi Valley and moved eastward; later, they converged to a convective fine line stretching along the associated cold front and sweeping from eastern Kentucky into Alabama. This precipitation event led to dozens of tornadoes, widespread rainstorms and straight-line winds reaching speeds of 78 mph. Prediction of the fine line, represented by the yellow line echo in the radar fields, is known to be very challenging.

pySTEPS predicts future radar fields of good sharpness but incurs large location error and fails to keep the shape of the line echo at 1 h ahead. PredRNN only provides an outline trend but the predictions are too blurry, losing the multiscale patterns useful for meteorologists to make forecasts. DGMR is able to preserve the convective details but suffers from unnatural cloud dissipation, yielding large location errors and underestimated intensities. Worse still, the shapes of the line predicted by DGMR are excessively distorted. Throughout the 3-h event, NowcastNet is the only method able to accurately predict the movement of the fine line and preserve the envelope of the rain area. The line echo covers intense rainfall (>32 mm h^−1^), for which NowcastNet achieves notably better CSI. NowcastNet also achieves the highest PSD at all wavelengths (that is, spatial scales), yielding sharp, consistent and multiscale nowcasts in reference to the ground truth.

**Fig. 3:**
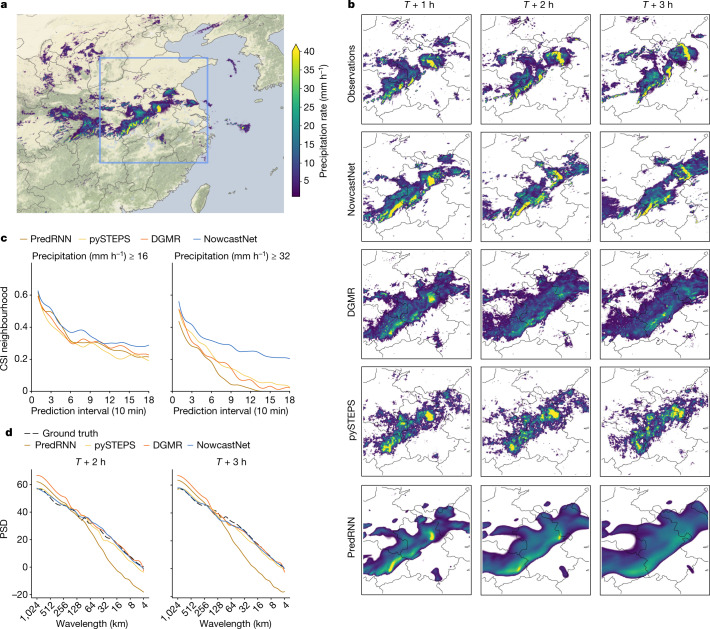
Case study of a precipitation event starting on 14 May 2021, with several convective cells and red rainstorm warnings in the Jianghuai area of China. NowcastNet is the only method able to predict the multiscale evolutions of the three convective cells over a longer time period. **a**, Geographic context for the predictions. **b**, A single prediction at *T* + 1 h, *T* + 2 h and *T* + 3 h lead times for different models. **c**, CSI neighbourhood at thresholds 16 mm h^−1^ and 32 mm h^−1^. **d**, PSD at different wavelengths. Images are zoomed in 768 km × 768 km to highlight local details. Precipitation data obtained from the China Meteorological Administration and maps produced with cartopy and Natural Earth. Source data

We investigate another precipitation event starting at 23:40 UTC on 14 May 2021 in the Jianghuai area of China (Fig. 3), for which several cities issued red rainstorm warnings. Three convective cells evolved differently. The first cell moved from the centre to the northeast, developing into a bow echo from a single-cell thunderstorm echo. The second cell was a squall line moving from the southwest to the middle, with the tail moving to the east. The third cell was in between and showed steady growth.

Subject to noncompliance of physical conservation laws, PredRNN and DGMR suffer from fast dissipation and fail to predict the evolution of any convective cell at a 2-h lead time. pySTEPS predicts the direction of the three cells but fails to predict the specific location or the shape change. By contrast, NowcastNet yields plausible nowcasts for the evolutions of the three cells at a 3-h lead time. Although the nowcasts of the squall line and the growing cell are still not perfect, they are useful for meteorologists. Quantitative results of NowcastNet in terms of CSI neighbourhood and PSD are substantially improved relative to the leading methods.

We inspect more weather events with extreme precipitation, convective initiation, light rainfall and typical processes in Extended Data Figs. 2–8 and Supplementary Figs. 2–5. High-resolution nowcasts of 2,048 km × 2,048 km are shown in Extended Data Figs. 9 and 10.

## Meteorologist evaluation

We evaluate the forecast value of different models for extreme-precipitation events by the meteorologist evaluation protocol from the UK Met Office^4^. For fairness, the China Meteorological Administration made a public invitation to senior meteorologists across China to participate in the evaluation. On the public website, experts can control the display of precipitation fields but the nowcasts of different models are shown anonymously and out of order. Finally, 62 expert meteorologists from the central and 23 provincial observatories completed the evaluation, each judging 15 test cases chosen randomly from the extreme-precipitation-event subsets. The USA and China subsets consist of 1,200 extreme events occurring over 93 days in 2021 and 50 days from April 2021 to June 2021, respectively. We note that, although judging the USA events by China meteorologists may incur some bias, we expect it to be relatively minor, as the global weather system shares underlying physical principles and the two countries share meteorological observations and technologies.

We augment the UK Met Office protocol by running two types of evaluation: posterior evaluation and prior evaluation. In the posterior evaluation, meteorologists were asked to objectively rank the forecasting value of the predictions of each model with reference to the future ground-truth observations. In the prior evaluation, meteorologists needed to subjectively rank the forecasting value given past radar series but without seeing the future ground truth. This protocol simulates the real scenario in which future observations are not accessible and meteorologists have to make an on-the-fly choice of which model is preferred for nowcasting.

**Fig. 4:**
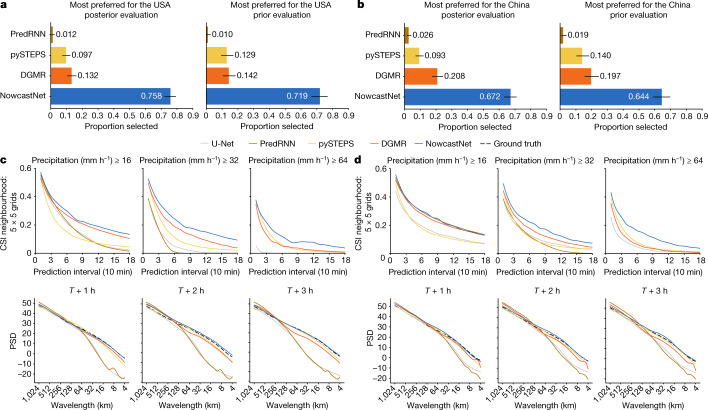
Meteorologist evaluation and quantitative evaluation of the USA and China datasets. **a**, Meteorologist evaluation for the USA events based on the first-choice preference of each model. Black horizontal lines indicate the Clopper–Pearson 95% confidence intervals. Meteorologists substantially preferred NowcastNet to other competitors (*n* = 62, *P* < 10^−4^). **b**, Meteorologist evaluation for the China events. Meteorologists substantially preferred NowcastNet to other competitors (*n* = 62, *P* < 10^−4^). **c**, Quantitative scores for the USA events in 2021. The first row shows CSI neighbourhood of different models at precipitation thresholds of 16 mm h^−1^, 32 mm h^−1^ and 64 mm h^−1^. The second row shows PSD over 1,024 km × 1,024 km predictions for different models at *T* + 1 h, *T* + 2 h and *T* + 3 h lead times. **d**, Quantitative scores for the China events from April 2021 to June 2021. Source data

The statistics of meteorologist evaluation are shown in Fig. 4a,b. In the posterior evaluation, NowcastNet was ranked as the first choice for 75.8% of the USA events ([72.1, 79.3]) and for 67.2% of the China events ([63.1, 71.1]). In the prior evaluation, NowcastNet was ranked as the first choice for 71.9% of the USA events ([66.6, 76.8]) and 64.4% of the China events ([58.9, 69.7]). The numbers in brackets are 95% confidence intervals. NowcastNet holds the highest meteorologist preference by providing skilful nowcasts that exhibit physical plausibility and multiscale features, whereas other models struggle.

## Quantitative evaluation

We provide a quantitative evaluation based on the results for CSI neighbourhood and PSD shown in Fig. 4c,d. The evaluation includes U-Net^30^, a common baseline for precipitation nowcasting. Adopting the importance-sampling protocol of DGMR^4^, we sample two subsets from the USA and China corpora, both representative of extreme-precipitation events. By CSI neighbourhood, NowcastNet produces more accurate nowcasts at higher rain rate (>16 mm h^−1^). By PSD, NowcastNet yields sharper nowcasts of more consistent variability in spectral characteristics to radar observations for a 3-h lead time. These quantities justify that NowcastNet is skilful for extreme-precipitation nowcasting, better able to predict precipitation patterns at both the mesoscale and the convective scale, while maintaining high accuracy of evolution prediction over a longer time period.

In Supplementary Figs. 10–17, we provide further quantitative evaluations under both uniform-sampling and importance-sampling protocols^4^.

## Conclusion

Precipitation nowcasting is a leading long-term goal of meteorological science. Although progress has been made, numerical weather-prediction systems are at present unable to provide skilful nowcasts for extreme-precipitation events that are needed for weather-dependent policymaking.

Much of the inherent difficulty of nowcasting stems from the multiscale and multiphysics problems arising in the atmosphere and the need to combine physical first principles with statistical-learning methods in a rigorous way. Our work addresses this challenge using an end-to-end optimization framework that combines physical-evolution schemes and conditional-learning methods. The resulting model, NowcastNet, provides physically plausible nowcasts with high resolution, long lead time and local details for extreme-precipitation events, for which existing methods struggle.

Much future work is needed to improve precipitation nowcasting skill. One direction is integration of more physical principles such as momentum conservation. Another direction is exploitation of more meteorological data such as satellite observations. We hope this work will inspire future research in these directions.

## Methods

Detailed explanations of the proposed model, as well as baselines, datasets and evaluations, are given here, with references to the Extended Data Figs. and Supplementary Information that add to the results provided in the main text.

### Model details

We describe NowcastNet with important details of the model architectures, the training methods and the hyperparameter tuning strategies. Ablation study of NowcastNet is available in Supplementary Information section A.

#### Evolution network

The 2D continuity equation modified for precipitation evolution^31^ is

2 $$\frac{\partial {\bf{x}}}{\partial t}+({\bf{v}}\cdot \nabla ){\bf{x}}={\bf{s}}.$$

Here **x**, **v** and **s** indicate radar fields of composite reflectivity, motion fields and intensity residual fields, respectively, and ∇ denotes the gradient operator. The tendency term (**v** ⋅ ∇)**x** reveals the mass leaving the system, which is the first-order approximation of the difference before and after the advection operation:

3 $$\frac{{\bf{x}}({\bf{p}}+\Delta t\cdot \Delta {\bf{v}},t+\Delta t)-{\bf{x}}({\bf{p}},t)}{\Delta t},$$

with **p** and *t* being the position and time, respectively. The residual field **s** shows the additive evolution mechanisms, such as the growth and decay of precipitation intensities. According to the continuity equation, the temporal evolution of precipitation can be modelled as a composition of advection by motion fields and addition by intensity residuals, which is the evolution operator we design for the evolution network. We use deep neural networks to simultaneously predict all these fields based on past radar observations, which enables nonlinear modelling capability for the complex precipitation evolution.

**Extended Data Fig. 1:**
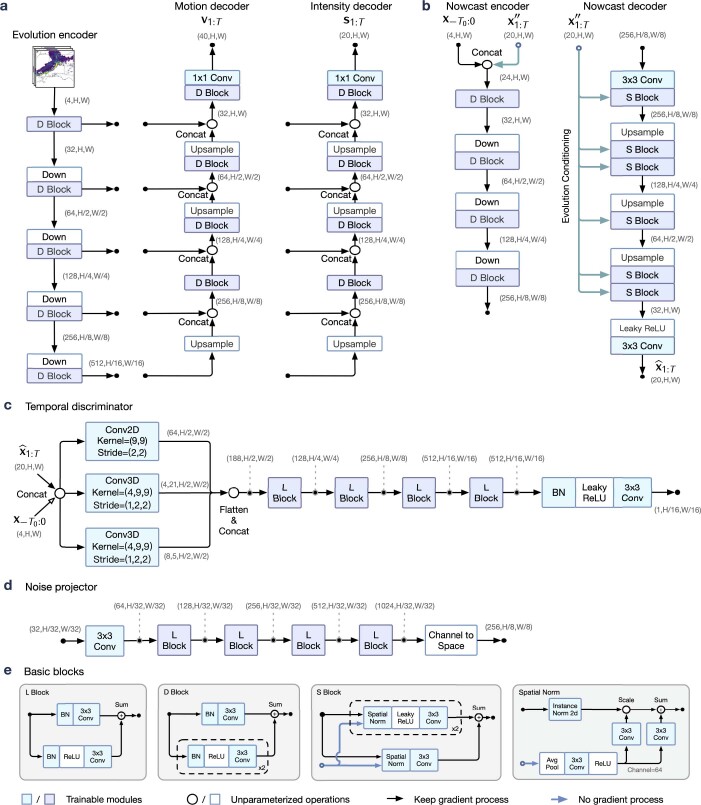
Architecture details of NowcastNet. **a**, Evolution network. **b**, Generative network. **c**, Temporal discriminator. **d**, Noise projector. **e**, Basic blocks. The input fields are of height *H* and width *W*. The convolutional layer uses (*N*,*N*)-kernel. Leaky ReLU is the leaky rectifier linear unit with negative slope of 0.2. BN is the batch normalization. Up and Down are bilinear interpolations to expand or reduce spatial size. Avg Pool is the spatial average pooling. Spatial Norm and Instance Norm are the normalizations applied within the spatially adaptive normalization to implement the physics-conditioning mechanism between the generative network and the evolution network.

The evolution network (Fig. 1b) takes as input past radar observations ${{\bf{x}}}_{-{T}_{0}:0}$ and predicts future radar fields ${{\bf{x}}}_{1:T}^{{\prime\prime} }$ at a 20-km scale based on a nonlinear, learnable evolution scheme we propose specifically in this article. The architecture details are described in Extended Data Fig. 1a. The backbone of the evolution network is a two-path U-Net^30^, which has a shared evolution encoder for learning context representations, a motion decoder for learning motion fields **v**_1:*T*_ and an intensity decoder for learning intensity residuals **s**_1:*T*_. The spectral normalization technique^32^ is applied in every convolution layer. In the skip connections of U-Net, all input and output fields are concatenated on the temporal dimension, that is, the channels in convolutional networks.

The evolution operator (Fig. 1c) is at the core of the evolution network. We use the backward semi-Lagrangian scheme as the advection operator. Because **v**_1:*T*_ is learnable, we directly set it as the departure offset of the semi-Lagrangian scheme. Also, because **s**_1:*T*_ is learnable, we directly use it to model the growth or decay of precipitation intensities. We take precipitation rate instead of radar reflectivity as the unit of radar field **x**, as this modification will not influence the physical nature of the evolution process. As applying bilinear interpolation for several steps will blur the precipitation fields, we opt for the nearest interpolation in the backward semi-Lagrangian scheme for computing ${{\bf{x}}}_{t}^{{\prime} }$. Yet, the nearest interpolation is not differentiable at **v**_1:*T*_. We resolve this gradient difficulty by using bilinear interpolation (bili) to advect ${\left({{\bf{x}}}_{t}^{{\prime} }\right)}_{{\rm{bili}}}$ from ${{\bf{x}}}_{t-1}^{{\prime\prime} }$, **v**_1:*T*_, and use ${\left({{\bf{x}}}_{t}^{{\prime} }\right)}_{{\rm{bili}}}$ to compute the accumulation loss for optimizing the motion fields. Then we use the nearest interpolation to compute ${{\bf{x}}}_{t}^{{\prime} }$ from ${{\bf{x}}}_{t-1}^{{\prime\prime} }$, **v**_1:*T*_, and compute the evolved field ${{\bf{x}}}_{t}^{{\prime\prime} }={{\bf{x}}}_{t}^{{\prime} }+{{\bf{s}}}_{t}$. After each round of the evolution operator, we detach the gradient between two consecutive time steps because the overall system is underdetermined. Meanwhile, the successive interpolation operations will make end-to-end optimization unstable, and detaching the gradient (stop gradient in Fig. 1c) will markedly improve the numerical stability^33^.

The objective function for training the evolution network comprises two parts. The first part is the accumulation loss, which is the sum of the weighted *L*_1_ distances between real observations and predicted fields:

4 $${J}_{{\rm{accum}}}=\mathop{\sum }\limits_{t=1}^{T}\left({L}_{{\rm{wdis}}}\left({{\bf{x}}}_{t},{\left({{\bf{x}}}_{t}^{{\prime} }\right)}_{{\rm{bili}}}\right)+{L}_{{\rm{wdis}}}\left({{\bf{x}}}_{t},{{\bf{x}}}_{t}^{{\prime\prime} }\right)\right).$$

In particular, the weighted distance has the following form:

5 $${L}_{{\rm{wdis}}}\left({{\bf{x}}}_{t},{{\bf{x}}}_{t}^{{\prime} }\right)={\left\Vert \left({{\bf{x}}}_{t}-{{\bf{x}}}_{t}^{{\prime} }\right)\odot {\bf{w}}\left({{\bf{x}}}_{t}\right)\right\Vert }_{1},$$

in which the pixel-wise weight *w*(*x*) = min(24, 1 + *x*) is taken from DGMR^4^. Because the rain rate approximately follows a log-normal distribution^17^, it is necessary to add weight to balance different rainfall levels. Otherwise, neural networks will only fit light-to-medium precipitation taking dominant ratio in the data and heavy precipitation will not be accounted for sufficiently. We follow DGMR^4^ and use a weight proportional to the rain rate and clip it at 24 for robustness to spuriously large values in radar observations.

The second part is the motion-regularization term in the form of gradient norm, which is motivated in part by the continuity equation and in part by the fact that large precipitation patterns tend to be longer lived than small ones^8^:

6 $${J}_{{\rm{motion}}}=\mathop{\sum }\limits_{t=1}^{T}\left({\parallel \nabla {{\bf{v}}}_{t}^{1}\odot \sqrt{{\bf{w}}({{\bf{x}}}_{t})}\parallel }_{2}^{2}+{\parallel \nabla {{\bf{v}}}_{t}^{2}\odot \sqrt{{\bf{w}}({{\bf{x}}}_{t})}\parallel }_{2}^{2}\right),$$

in which ${{\bf{v}}}_{t}^{1}$ and ${{\bf{v}}}_{t}^{2}$ are the two components of the motion fields. The gradient of the motion fields ∇**v** is computed approximately with the Sobel filter^24^:

7 $${\partial }_{1}{\bf{v}}\approx \left(\begin{array}{ccc}1 & 0 & -1\\ 2 & 0 & -2\\ 1 & 0 & -1\end{array}\right)\,\ast \,{\bf{v}},\qquad {\partial }_{2}{\bf{v}}\approx \left(\begin{array}{ccc}\,1 & \,2 & \,1\\ \,0 & \,0 & \,0\\ -1 & -2 & -1\end{array}\right)\,\ast \,{\bf{v}},$$

in which ⁎ denotes the 2D convolution operator in the spatial dimension.

Overall, the objective for training the evolution network (Fig. 1b) is

8 $${J}_{{\rm{evolution}}}={J}_{{\rm{accum}}}+\lambda {J}_{{\rm{motion}}}\,.$$

During training, we sample the radar fields with 256 × 256 spatial size as the input. On both the USA and China datasets, we fix input length *T*_0_ = 9 and set output length *T* = 20 for training and take the first 18 predicted fields for evaluation. Note that increasing *T*_0_ does not provide substantial improvements and *T*_0_ ≥ 4 is sufficient. The tradeoff hyperparameter *λ* is set as 1 × 10^−2^. We use the Adam optimizer^34^ with a batch size of 16 and an initial learning rate of 1 × 10^−3^, and train the evolution network for 3 × 10^5^ iterations, during which we decay the learning rate to 1 × 10^−4^ at the 2 × 10^5^th iteration.

#### Generative network

Conditioning on the evolution network predictions ${{\bf{x}}}_{1:T}^{{\prime\prime} }$, the generative network takes as input the past radar observations ${{\bf{x}}}_{-{T}_{0}:0}$ and generates from latent random vectors **z** for the final predicted precipitation fields ${\hat{{\bf{x}}}}_{1:T}$ at a 1–2-km scale. The backbone of the generative network is a U-Net encoder–decoder structure, with architecture details shown in Extended Data Fig. 1b. The nowcast encoder has the identical structure as the evolution encoder (Extended Data Fig. 1a), which takes as input the concatenation of ${{\bf{x}}}_{-{T}_{0}:0}$ and ${{\bf{x}}}_{1:T}^{{\prime\prime} }$. The nowcast decoder is a different convolutional network, which takes as input the contextual representations from the nowcast encoder, along with the transformation of the latent Gaussian vector **z**. The designs of D Block, S Block and Spatial Norm heavily used in the generative network are elaborated in Extended Data Fig. 1e.

The noise projector transforms the latent Gaussian vector **z** to the same spatial size as the contextual representations from the nowcast encoder, as elaborated in Extended Data Fig. 1d. For each forward pass, each element of **z** is independently sampled from the standard Gaussian ${\mathcal{N}}(0,1)$. Then **z** is transformed by the noise projector into a tensor with one-eighth the height and width of input radar observations.

The physics-conditioning mechanism to fuse the generative network and the evolution network is implemented by applying the spatially adaptive normalization^20^ to each convolutional layer of the nowcast decoder (Extended Data Fig. 1b,e). First, each channel of the nowcast decoder is normalized by a parameter-free instance-normalization module^35^. Then the evolution network predictions ${{\bf{x}}}_{1:T}^{{\prime\prime} }$ are resized to a compatible spatial size and then concatenated to the nowcast decoder at the corresponding layer through average pooling. Finally, a two-layer convolutional network transforms the resized predictions into new mean and variance for each channel of the nowcast decoder, ensuring not to distort the spatial-coherent features from the evolution network predictions ${{\bf{x}}}_{1:T}^{{\prime\prime} }$. Through the physics-conditioning mechanism, the generative network is adaptively informed by the physical knowledge learned with the evolution network, while resolving the inherent conflict between physical-evolution and statistical-learning regimes.

Conditioning on the evolution network predictions at a 20-km scale, the generative network is needed to further generate convective details at a 1–2-km scale through training on a temporal discriminator *D* (Extended Data Fig. 1c). The temporal discriminator takes as input real radar observations ${{\bf{x}}}_{1:T}$ and final predicted fields ${\hat{{\bf{x}}}}_{1:T}$ and outputs scores of how likely they are being real or fake. At its first layer, the inputs are processed by 3D convolution layers with several kernel sizes at the temporal dimension from 4 to the full horizon. Then the multiscale features are concatenated and feedforwarded to subsequent convolutional layers with spectral normalization^32^ applied in each layer. The objective for training the temporal discriminator is

9 $${J}_{{\rm{d}}{\rm{i}}{\rm{s}}{\rm{c}}}={L}_{{\rm{c}}{\rm{e}}}(D({{\bf{x}}}_{1:T}),1)+{L}_{{\rm{c}}{\rm{e}}}(D({\hat{{\bf{x}}}}_{1:T}),0),$$

with *L*_ce_ being the cross-entropy loss. Within a two-player minimax game, the nowcast decoder of the generative network is trained to confuse the temporal discriminator by minimizing the adversarial loss modified by^21^

10 $${J}_{{\rm{a}}{\rm{d}}{\rm{v}}}={L}_{{\rm{c}}{\rm{e}}}(D({\hat{{\bf{x}}}}_{1:T}),1).$$

The gradients backpropagate through ${\hat{{\bf{x}}}}_{1:T}$, first to the nowcast decoder and then to the nowcast encoder of the generative network, leading it to predict realistic multiscale fields with convective-scale details.

We take the idea of generative ensemble forecasting from DGMR^4^ and predict a group of precipitation fields ${\hat{{\bf{x}}}}_{1:T}^{{{\bf{z}}}_{i}}$ from several latent inputs **z**_1:*k*_, with *k* being the number of ensemble members. Then we aggregate the *k* predictions ${\hat{{\bf{x}}}}_{1:T}^{{{\bf{z}}}_{i}}$ and real fields **x**_1:*T*_ respectively by a max-pooling layer *Q* in the spatial dimension, with kernel size and stride set as 5 and 2, correspondingly. On the basis of ensemble forecasts, the pool regularization is defined as the weighted distance between spatial-pooled observations and the mean of *k* spatial-pooled predictions

11 $${J}_{{\rm{p}}{\rm{o}}{\rm{o}}{\rm{l}}}={L}_{{\rm{w}}{\rm{d}}{\rm{i}}{\rm{s}}}\left(Q({{\bf{x}}}_{1:T}),\frac{1}{k}\mathop{\sum }\limits_{i=1}^{k}Q({\hat{{\bf{x}}}}_{1:T}^{{{\bf{z}}}_{i}})\right).$$

Overall, the objective for training the generative network (Fig. 1a) is

12 $${J}_{{\rm{generative}}}=\beta {J}_{{\rm{adv}}}+\gamma {J}_{{\rm{pool}}}\,.$$

We set the number of ensemble members as *k* = 4, adversarial loss weight *β* = 6 and pool-regularization weight *γ* = 20. Similar to the evolution network, we set input length *T*_0_ = 9 and output length *T* = 20. We use the Adam optimizer^34^ with a batch size of 16 and an initial learning rate of 3 × 10^−5^ for the nowcast encoder, the nowcast decoder and the temporal discriminator and train the generative network for 5 × 10^5^ iterations.

#### Transfer learning

NowcastNet is a foundational model for skilful precipitation nowcasting. A large-scale dataset will help NowcastNet be more apt at learning physical evolution and chaotic dynamics of the precipitation processes. Therefore, in countries or regions with intricate atmosphere processes but without sufficient radar observations, we use the transfer learning strategy^27^, a de facto way to reusing knowledge from pre-trained foundational models. Given a pre-trained NowcastNet model, we use the objectives *J*_evolution_ and *J*_generative_ to fine-tune its evolution network and generative network through decoupled backpropagation, which detaches the gradients between *J*_evolution_ and *J*_generative_. As the physical knowledge behind the precipitation is universal and transferable across the world, we decrease the learning rate of the evolution network as one-tenth that for the generative network to avoid forgetting^36^ of physical knowledge. We pre-train a NowcastNet model on a large-scale dataset and fine-tune it to a small-scale dataset with the Adam optimizer^34^, but only for 2 × 10^5^ iterations.

#### Hyperparameter tuning

We use the mean of CSI neighbourhood (CSIN) over all prediction time steps at the rain levels of 16 mm h^−1^, 32 mm h^−1^ and 64 mm h^−1^ when tuning the hyperparameters of the evolution network. We compute the criterion for hyperparameter tuning as the average of the quantities, $\frac{{{\rm{CSIN}}}_{16}+{{\rm{CSIN}}}_{32}+{{\rm{CSIN}}}_{64}}{3}$. When tuning the hyperparameters of the generative network, we use the two main evaluation metrics, CSI neighbourhood and PSD. For each model with different hyperparameters, we first ensure that the PSD of the model is no worse than that of pySTEPS. Then we use the average CSI neighbourhood criterion $\frac{{{\rm{CSIN}}}_{16}+{{\rm{CSIN}}}_{32}+{{\rm{CSIN}}}_{64}}{3}$ to determine the final hyperparameters.

### Baselines

We describe the four baselines used in the comparative study. There is a rich literature of relevant work and we discuss them as further background in Supplementary Information section E.

#### DGMR

DGMR is a state-of-the-art method for precipitation nowcasting, recognized by expert meteorologists. We genuinely reproduce it taking exactly the same architecture and training settings described in ref. ^4^ and the released model files available at https://github.com/deepmind/deepmind-research/tree/master/nowcasting, with the quantitative and qualitative results to match those reported in the original paper. We set the number *k* of ensemble members as 4 during training, which is the same as NowcastNet.

#### PredRNN-V2

We consider PredRNN-V2 (ref. ^13^), the latest version of PredRNN^37^ with a four-layer convolutional-recurrent network, deployed at the China Meteorological Administration for operational nowcasting. We cut radar fields into 4 × 4 patches and unfold the patches as the channel dimension, which efficiently balances the computation cost and forecasting skill. Reverse scheduled sampling with an exponential increasing strategy is applied in the first 5 × 10^4^ iterations.

#### U-Net

We use the improved version proposed by Ravuri et al.^4^, which adds a residual structure in each block of the vanilla U-Net^30^, along with a loss weighted by precipitation intensity, and predicts all fields in a single forward pass.

#### pySTEPS

We use the pySTEPS implementation from ref. ^9^, following the default settings available at https://github.com/pySTEPS/pysteps.

All deep-learning models, including NowcastNet, DGMR, PredRNN-V2 and U-Net, are trained on the USA dataset (years 2016–2020) by the Adam optimizer with a batch size of 16 for 5 × 10^5^ iterations and transferred to the China dataset by fine-tuning for 2 × 10^5^ iterations. For all models under evaluation, we establish a fair comparison by using the same weighting scheme *w*(*x*) in the weighted distance *L*_wdis_ and the same sampling strategy of training data. Both the weighting scheme and the sampling strategy are taken from DGMR^4^.

### Datasets

**Extended Data Fig. 2:**
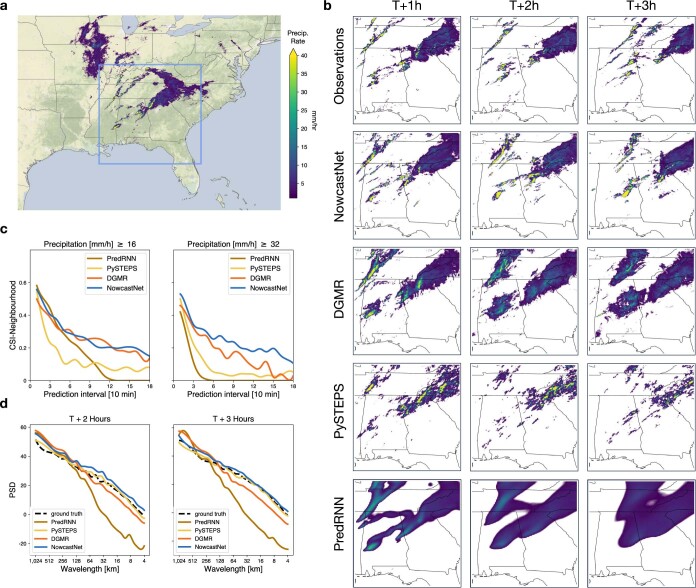
Case study of a precipitation event starting at 23:50 UTC on 25 March 2021, with a tornado outbreak across several states of Alabama, Georgia and Tennessee. NowcastNet provides the only results that have forecast skills on high-intensity precipitation and show the sharp structures of several supercells for the 3-h horizon. **a**, Geographic context for the predictions. **b**, A single prediction at *T* + 1 h, *T* + 2 h and *T* + 3 h lead times for different models. **c**, CSI neighbourhood at thresholds 16 mm h^−1^ and 32 mm h^−1^. **d**, PSD at different wavelengths. Images are zoomed in 768 km × 768 km to highlight local details. Precipitation data obtained from the MRMS^26^ dataset and maps produced with cartopy and Natural Earth.

**Extended Data Fig. 3:**
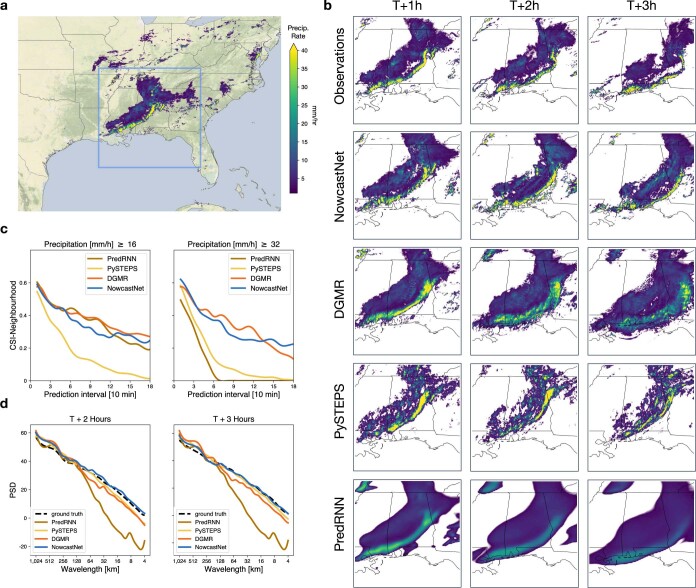
Case study of a precipitation event starting at 23:10 UTC on 4 May 2021, with a massive squall line that swept across several states in southeast USA. Compared with other baselines, NowcastNet is the only model that simultaneously keeps the shape and intensity of the squall line. **a**, Geographic context for the predictions. **b**, A single prediction at *T* + 1 h, *T* + 2 h and *T* + 3 h lead times for different models. **c**, CSI neighbourhood at thresholds 16 mm h^−1^ and 32 mm h^−1^. **d**, PSD at different wavelengths. Images are zoomed in 768 km × 768 km to highlight local details. Precipitation data obtained from the MRMS^26^ dataset and maps produced with cartopy and Natural Earth.

**Extended Data Fig. 4:**
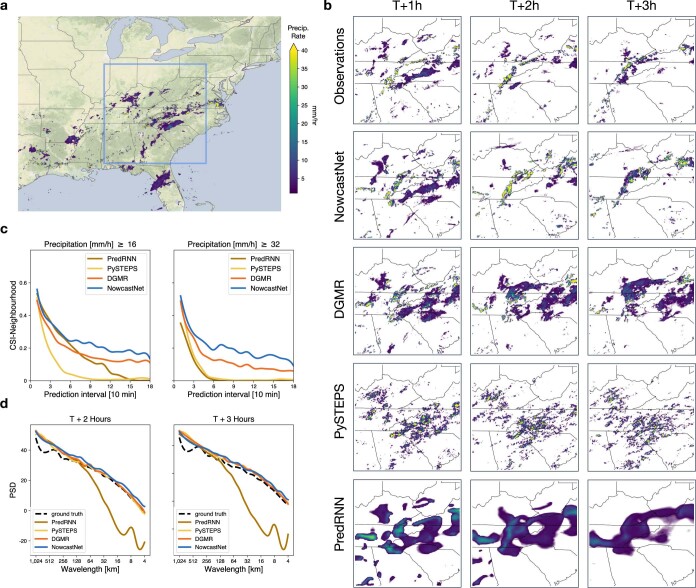
Case study of a precipitation event starting at 23:20 UTC on 14 August 2021, with widespread convective weather occurring over eastern Tennessee. In the predictions of the four models, only NowcastNet provides clear nowcast of the initiation and the dissipation of the storm line. **a**, Geographic context for the predictions. **b**, A single prediction at *T* + 1 h, *T* + 2 h and *T* + 3 h lead times for different models. **c**, CSI neighbourhood at thresholds 16 mm h^−1^ and 32 mm h^−1^. **d**, PSD at different wavelengths. Images are zoomed in 768 km × 768 km to highlight local details. Precipitation data obtained from the MRMS^26^ dataset and maps produced with cartopy and Natural Earth.

**Extended Data Fig. 5:**
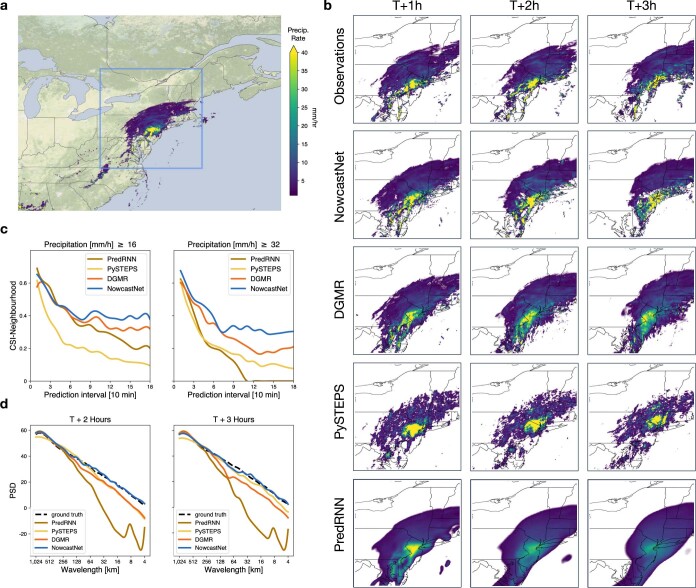
Case study of a precipitation event starting at 22:30 UTC on 1 September 2021, with the remnants of Hurricane Ida approaching northeastern USA. NowcastNet provides better predictions on the evolution of high-intensity precipitation and is able to keep the contour of the cyclone system across 3 h. **a**, Geographic context for the predictions. **b**, A single prediction at *T* + 1 h, *T* + 2 h and *T* + 3 h lead times for different models. **c**, CSI neighbourhood at thresholds 16 mm h^−1^ and 32 mm h^−1^. **d**, PSD at different wavelengths. Images are zoomed in 768 km × 768 km to highlight local details. Precipitation data obtained from the MRMS^26^ dataset and maps produced with cartopy and Natural Earth.

**Extended Data Fig. 6:**
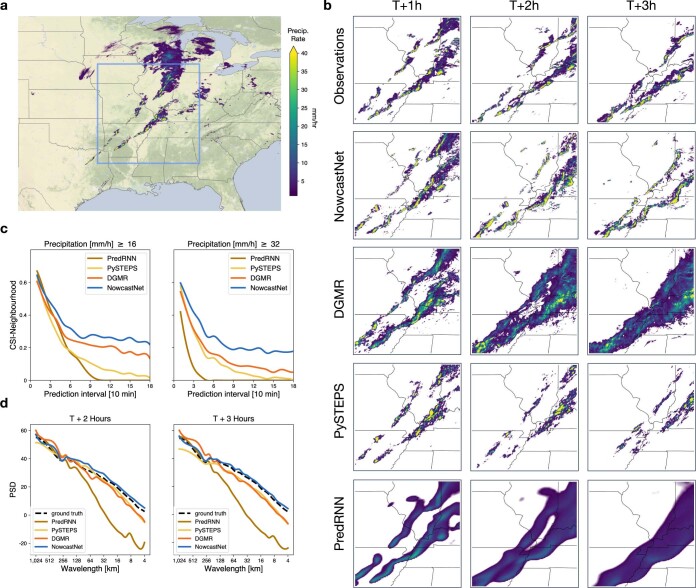
Case study of a precipitation event starting at 03:50 UTC on 11 December 2021, with a tornado outbreak that hit the central area around Tennessee. NowcastNet gives detailed predictions on the movements and intensities of the two storms and yields a more accurate description of the motions of several supercells. **a**, Geographic context for the predictions. **b**, A single prediction at *T* + 1 h, *T* + 2 h and *T* + 3 h lead times for different models. **c**, CSI neighbourhood at thresholds 16 mm h^−1^ and 32 mm h^−1^. **d**, PSD at different wavelengths. Images are zoomed in 768 km × 768 km to highlight local details. Precipitation data obtained from the MRMS^26^ dataset and maps produced with cartopy and Natural Earth.

**Extended Data Fig. 7:**
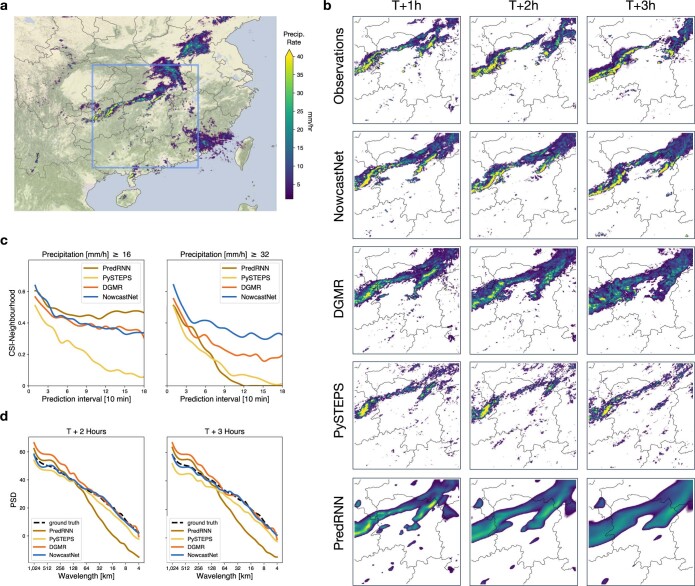
Case study of a precipitation event starting at 06:50 UTC on 3 May 2021, with a squall-line system causing hail orange alert at the western Hunan province of China. NowcastNet provides more accurate predictions on the formation and movement of the squall line. **a**, Geographic context for the predictions. **b**, A single prediction at *T* + 1 h, *T* + 2 h and *T* + 3 h lead times for different models. **c**, CSI neighbourhood at thresholds 16 mm h^−1^ and 32 mm h^−1^. **d**, PSD at different wavelengths. Images are zoomed in 768 km × 768 km to highlight local details. Precipitation data obtained from the China Meteorological Administration and maps produced with cartopy and Natural Earth.

**Extended Data Fig. 8:**
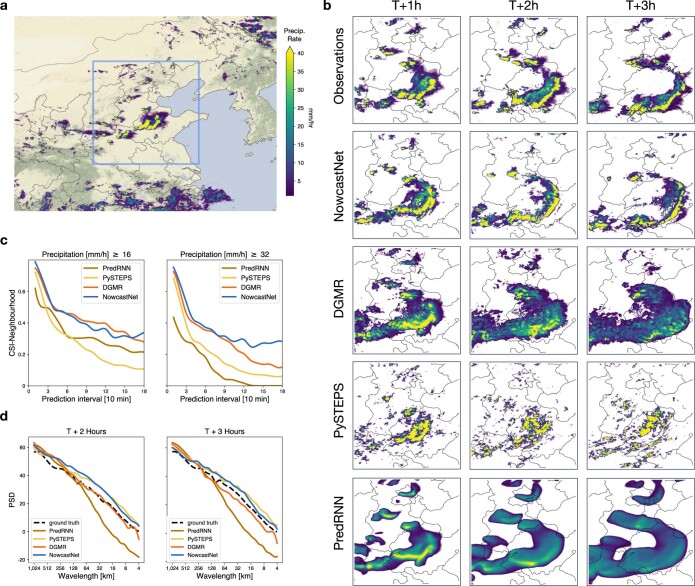
Case study of a precipitation event starting at 07:50 UTC on 30 June 2021, with a squall line that developed quickly and swept across the Shandong province of China, causing several red warnings. NowcastNet provides the only sharp and meticulous predictions on the shape and the location of the squall-line-developing system. **a**, Geographic context for the predictions. **b**, A single prediction at *T* + 1 h, *T* + 2 h and *T* + 3 h lead times for different models. **c**, CSI neighbourhood at thresholds 16 mm h^−1^ and 32 mm h^−1^. **d**, PSD at different wavelengths. Images are zoomed in 768 km × 768 km to highlight local details. Precipitation data obtained from the China Meteorological Administration and maps produced with cartopy and Natural Earth.

Two large-scale, high-resolution datasets of composite radar observations from the USA and China are used throughout the experiments. The evaluation metrics are described in Supplementary Information section B. More case studies of representative precipitation events and quantitative results of overall performance are available in Extended Data Figs. 2–8 and Supplementary Information sections C and D.

#### USA dataset

The USA dataset consists of radar observations from the MRMS system^26,38^, collected over the USA. The radar composites cover the area from 20 °N to 55 °N in the south–north direction and 130 °W to 60 °W in the east–west direction. The spatial grid of the composites is 3,500 × 7,000, with a resolution of 0.01° per grid. The missing values on the composites are assigned negative values, which can mask unconcerned positions during evaluation. We use radar observations collected for a 6-year time range from 2016 to 2021, in which the training set covers years 2016–2020 and the test set covers the year 2021. We follow the strategy used in ref. ^4^ such that the radar observations from the first day of each month in the training set are included in the validation set. To trade off computational cost and forecasting skill, we set the temporal resolution as 10 min and downscale the spatial size of radar fields to half of the original width and height, which will keep the most of the convective-scale details. We cap the rain rates at the value of 128 mm h^−1^.

#### China dataset

The China dataset includes radar observations collected over China by the China Meteorological Administration. The radar composites cover the area from 17° N to 53° N in the south–north direction and 96° E to 132° E in the east–west direction, with a coverage of the middle and east of China. The spatial grid of the composites is 3,584 × 3,584, with a resolution of 0.01° per grid. Similar to the USA dataset, the missing values are replaced by negative values. We use radar observations collected for a nearly 2-year time range from 1 September 2019 to 30 June 2021. Data from 1 September 2019 to 31 March 2021 are taken as the training set, whereas those from 1 April 2021 to 30 June 2021 are taken as the test set. We follow the strategy used in ref. ^4^ such that the radar observations from the first day of each month in the training set are included in the validation set. Notably, the test period covers the flood season when extreme precipitation and rainstorms are frequent in China. We set the temporal resolution, spatial size and rain-rate threshold exactly the same as the USA dataset.

#### Data preparation

We construct the training set and test set for each dataset using an importance-sampling strategy^4^ to increase the ratio of radar series with heavy precipitation. We first crop the full-frame series into smaller spatiotemporal size. For the training set, we cut the series into crops of spatial size 256 × 256 and temporal size 270 min with offsets of 32 in the vertical and horizontal directions. For the test set, we cut the series into crops of spatial size 512 × 512 and temporal size 270 min with offsets of 32 in the vertical and horizontal directions. Then we give each crop an acceptance probability,

13 $$\Pr ({{\bf{x}}}_{-{T}_{0}:T})=\mathop{\sum }\limits_{t=-{T}_{0}}^{T}{\left\Vert {\bf{g}}({{\bf{x}}}_{t})\right\Vert }_{1}+{\epsilon },$$

which is the sum of radar fields for all grids and all time steps on this crop, and *ϵ* is a small constant. As done in DGMR^4^, for the training set, we set g(*x*) = 1 − e^−*x*^ on each grid with a valid value and g(*x*) = 0 on each grid with a missing value. We use hierarchical sampling during training, by first sampling the full-frame series and then sampling the crop series. To evaluate the forecasting skill of different models on extreme-precipitation events, we define g(*x*) = *x* for the test set. The test set is sampled in advance and kept unchanged throughout evaluation. As our goal is skilful nowcasting of extreme precipitation, this importance-sampling strategy is biased towards weather events with a larger proportion of heavy precipitation.

We also use the uniform-sampling protocol such that all light-to-heavy precipitation can be equally evaluated. In this protocol, the crops in the test set are sampled uniformly from all spatial and temporal ranges. Because the uniformly sampled series usually have scarce precipitation, we enlarge the dataset size to 288,000 for the USA case and 120,000 for the China case, three times larger than the importance-sampled test datasets. The quantitative results under this protocol are available in Supplementary Figs. 10 and 11.

### Evaluation

We perform a meteorologist evaluation as a cognitive assessment task and a quantitative evaluation using operational verification measures.

#### Meteorologist evaluation

To construct the test subsets representative of extreme-precipitation events for expert meteorologist evaluation, we first sample a new test set that contains the crops with spatial size of 512 × 512 using the same strategy detailed in the previous section. After this test set is sampled, we rank the crops by the sum of rain rate on all grids with rate higher than a threshold of 20 mm h^−1^. This is the threshold of heavy rainfall used in operational practice by the China Meteorological Administration. We take the top 1,200 events as the subset for expert meteorologist evaluation. Because the test events are fewer, we change the strategy to ranking all events by the proportion of grids with a rate higher than 20 mm h^−1^, which include extreme precipitation with very high probability, while ensuring the temporal diversity. On all crops in this test subset, all models take as input the fields of spatial size 512 × 512, and the central 384 × 384 area of the predicted fields are zoomed in to highlight the convective details.

To enable a professional, transparent and fair meteorologist evaluation, the China Meteorological Administration issued a public announcement to all provincial meteorological observatories, inviting senior meteorologists to participate in the evaluation as volunteers. The announcement states the content, goal and how-to of the expert evaluation, and specifically clarifies that the evaluation results will only be used anonymously for the scientific research but not for the skill test of meteorologists or other purposes. Operationally, we build an anonymous website for the meteorologist evaluation. Each expert logs in to the website using an automatically generated user account with password protection to perform the evaluation anonymously, without being informed of any model information. In the posterior evaluation, we show real radar observations in the past and future horizons and the model predictions anonymously in random order for each event, whereas in the prior evaluation, we only show the real radar observations in the past. Meteorologists can play the video, navigate the progress bar to deliberately observe cloud evolution or arbitrarily stop the video at a certain time step for a meticulous comparison of the forecasting skill and value of all models.

#### Quantitative evaluation

Evaluation with commonly used quantitative metrics involves comparing the difference between ground truths and model predictions on the crops in the test set. Each model outputs 18 future frames of precipitation fields given nine past frames of radar observations, whereas pySTEPS is given four past frames. Similar to the evaluation protocol of DGMR^4^, the input spatial size is set as 512 × 512 for computing the PSD metric and as 256 × 256 for computing the other metrics. We apply the central-cropping technique, which crops 64 × 64 grid cubes from the central area of the 18 predicted frames, along with the corresponding ground truths. The PSD metric is directly computed on the 512 × 512 precipitation fields, whereas the other metrics are computed between the predicted and ground-truth cubes. The central cropping can eliminate the boundary influence and reduce the computation cost^4^. For methods with ensemble-forecasting ability, including NowcastNet, DGMR and pySTEPS, we set the number *k* of ensemble members as 4 for computing specific quantitative measures.

## Online content

Any methods, additional references, Nature Portfolio reporting summaries, source data, extended data, supplementary information, acknowledgements, peer review information; details of author contributions and competing interests; and statements of data and code availability are available at 10.1038/s41586-023-06184-4.

## Supplementary information

Supplementary Information The supplementary information consists of five sections: section A elaborates ablation studies of NowcastNet; section B describes the evaluation metrics; section C shows demonstrations of further precipitation events; section D gives further quantitative results; section E reviews the related works.

Supplementary Video 1 Video of precipitation nowcasting on the event shown in Fig. 2. The precipitation event started on 11 December 2021, with a large convective fine line and a tornado outbreak in eastern USA. Precipitation data obtained from the MRMS dataset and maps produced with cartopy and Natural Earth.

Supplementary Video 2 Video of precipitation nowcasting on the event shown in Fig. 3. The precipitation event started on 14 May 2021, with several convective cells and red rainstorm warnings in the Jianghuai area of China. Precipitation data obtained from the China Meteorological Administration and maps produced with cartopy and Natural Earth.

Supplementary Video 3 Video of precipitation nowcasting on the event shown in Extended Data Fig. 2. The precipitation event started at 23:50 UTC on 25 March 2021, with a tornado outbreak across several states of Alabama, Georgia and Tennessee. Precipitation data obtained from the MRMS dataset and maps produced with cartopy and Natural Earth.

Supplementary Video 4 Video of precipitation nowcasting on the event shown in Extended Data Fig. 3. The precipitation event started at 23:10 UTC on 4 May 2021, with a massive squall line that swept across several states in southeast USA. Precipitation data obtained from the MRMS dataset and maps produced with cartopy and Natural Earth.

Supplementary Video 5 Video of precipitation nowcasting on the event shown in Extended Data Fig. 4. The precipitation event started at 23:20 UTC on 14 August 2021, with widespread convective weather occurring over eastern Tennessee. Precipitation data obtained from the MRMS dataset and maps produced with cartopy and Natural Earth.

Supplementary Video 6 Video of precipitation nowcasting on the event shown in Extended Data Fig. 5. The precipitation event started at 22:30 UTC on 1 September 2021, with the remnants of Hurricane Ida approaching northeastern USA. Precipitation data obtained from the MRMS dataset and maps produced with cartopy and Natural Earth.

Supplementary Video 7 Video of precipitation nowcasting on the event shown in Extended Data Fig. 6. The precipitation event started at 03:50 UTC on 11 December 2021, with a tornado outbreak that hit the central area around Tennessee. Precipitation data obtained from the MRMS dataset and maps produced with cartopy and Natural Earth.

Supplementary Video 8 Video of precipitation nowcasting on the event shown in Extended Data Fig. 7. The precipitation event started at 06:50 UTC on 3 May 2021, with a squall-line system causing hail orange alert at the western Hunan Province of China. Precipitation data obtained from the China Meteorological Administration and maps produced with cartopy and Natural Earth.

Supplementary Video 9 Video of precipitation nowcasting on the event shown in Extended Data Fig. 8. The precipitation event started at 07:50 UTC on 30 June 2021, with a squall line that developed quickly and swept across the Shandong Province of China, causing several red warnings. Precipitation data obtained from the China Meteorological Administration and maps produced with cartopy and Natural Earth.

## Figures and Tables

**Extended Data Fig. 9:**
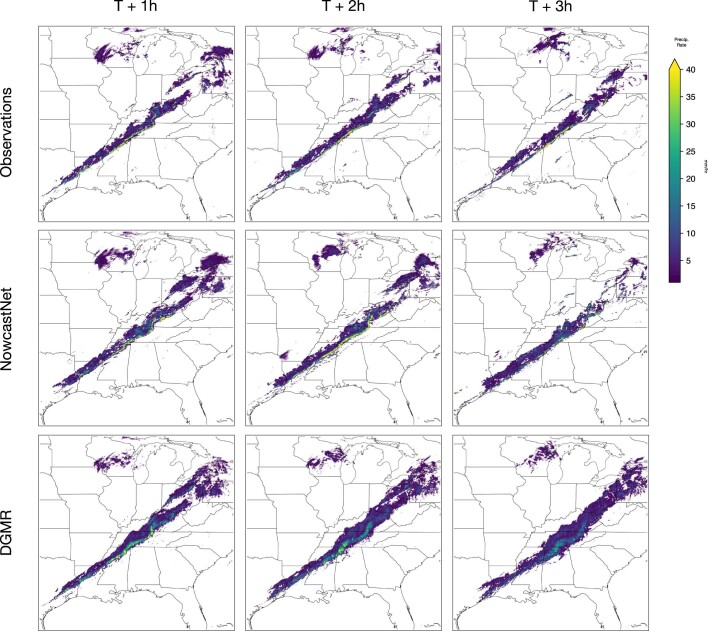
High-resolution precipitation nowcasting with spatial range of 2,048 km × 2,048 km. The precipitation event started at 09:30 UTC on 11 December 2021 in eastern and central USA, with a widespread convective fine line accompanied by a tornado outbreak. NowcastNet is better able to predict the convective fine-line evolutions and details for a longer time period. Precipitation data obtained from the MRMS^26^ dataset and maps produced with cartopy and Natural Earth.

**Extended Data Fig. 10:**
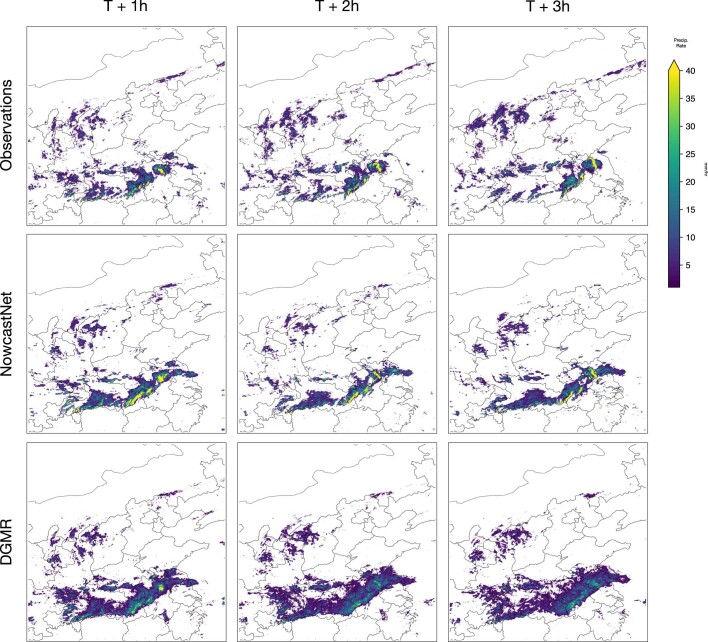
High-resolution precipitation nowcasting with spatial range of 2,048 km × 2,048 km. The precipitation event started at 23:40 UTC on 14 May 2021 in central and eastern China, with several convective cells causing red rainstorm warnings. NowcastNet is the only method that is able to predict the multiscale evolutions of the three convective cells over a longer time period. Precipitation data obtained from the China Meteorological Administration and maps produced with cartopy and Natural Earth.

## Data Availability

The processed radar data that support the findings of this study are available on the Tsinghua Cloud with the accession code ‘nowcast’; see https://cloud.tsinghua.edu.cn/d/b9fb38e5ee7a4dabb2a6. A smaller dataset with the code for exploratory analysis is available on Code Ocean at 10.24433/CO.0832447.v1. The MRMS data that support the training of the nowcasting models for the USA weather system are available with agreement from the NOAA at https://www.nssl.noaa.gov/projects/mrms or contact the MRMS data teams using mrms@noaa.gov. The radar data that support the training of the nowcasting models for the China weather system are available from the China Meteorological Administration but restrictions apply to the availability of these data, which were used under license for the current study and so are not publicly available. Data are available from the authors on reasonable request and with permission of the China Meteorological Administration. Source data are provided with this paper.

## Source data

Source Data Fig. 2
Source Data Fig. 3
Source Data Fig. 4

## Code availability

We rely on PyTorch (https://pytorch.org) for deep model training and cartopy (https://scitools.org.uk/cartopy) for geospatial data processing. We use specialized open-source tools for pySTEPS (https://pysteps.github.io), DGMR (https://github.com/deepmind/deepmind-research/tree/master/nowcasting), PredRNN-V2 (https://github.com/thuml/predrnn-pytorch) and SPADE (https://github.com/NVlabs/SPADE). The code of NowcastNet and the pre-trained neural-network weights are available on Code Ocean (10.24433/CO.0832447.v1).
