# Supplementary material for: Skilful nowcasting of extreme precipitation with NowcastNet
Source: Nature. 2023 Jul 5;619(7970):526–32. doi: 10.1038/s41586-023-06184-4 (PMC10356617; doi:10.1038/s41586-023-06184-4)
Supplement: Supplementary file 1 — The supplementary information consists of five sections: section A elaborates ablation studies of NowcastNet; section B describes the evaluation metrics; section C shows demonstrations of further precipitation events; section D gives further quantitative results; section E reviews the related works. [file 41586_2023_6184_MOESM1_ESM.pdf]

nature portfolio

https://doi.org/10.1038/s41586-023-06184-4

Supplementary information

Skilful nowcasting of extreme precipitation with NowcastNet

In the format provided by the authors and unedited

Contents

A Ablation study of NowcastNet **2**
A.1 Physics-conditioning mechanism 2
A.2 Major designs of evolution network 2

B Evaluation metrics **5**

C Additional precipitation events **5**

D Additional quantitative results **15**

E Related work **24**

A Ablation study of NowcastNet

NowcastNet is a physics-conditional deep generative model, comprised of the generative network and the evolution network. We provide an extensive ablation study to investigate the effect and behavior of different modules in NowcastNet, with a focus on the major designs of the evolution network and the physics-conditioning mechanism between the generative network and the evolution network. The ablation results in reference to the ground truth observations and PySTEPS are shown in Supplementary Fig. 1.

A.1 Physics-conditioning mechanism

We first inspect the physics-conditioning mechanism in NowcastNet, which adaptively controls the integration of physical knowledge from the evolution network into the generative network. Besides the default conditioning mechanism in NowcastNet, we investigate another two natural alternatives: weak conditioning and no conditioning.

In the weak conditioning model, we remove from NowcastNet the evolution conditioning module (Fig. 1a), which enables fuller integration of the evolution network predictions to multiple layers of the generative network; Namely, we simply take the predictions as another input to the U-Net based nowcast decoder. In principle, U-Net employs shortcut connections from the bottom layers to the top layers to deliver multiscale features, making the generative network weakly conditioned on the evolution network. As shown in Supplementary Fig. 1, while the location of the predicted squall line by the weak conditioning model (3rd row) is relatively consistent with the original NowcastNet, the intensity of the squall line is not kept after two hours. Worse still, there are undesired extra noises in the predictions that are out of the scope of the evolution network predictions.

In the no conditioning model, we remove the whole evolution network from NowcastNet, but keep all other training settings unchanged. As shown in Supplementary Fig. 1, the no conditioning model (4th row) exhibits a large tendency of dissipating the heavy precipitation and violating the cloud dynamics, thereby generating unusable predictions after one hour. This is a strong evidence that the physics-conditioning on the evolution network informed by physical knowledge is important to make NowcastNet skillful for extreme precipitation nowcasting.

A.2 Major designs of evolution network

We inspect the major designs of the evolution network, and justify that each design is crucial to the skillful performance. As shown in Supplementary Fig. 1, by comparing NowcastNet (2nd row) and the evolution network (5th row), we can clearly observe that the evolution network can only produce mesoscale nowcasts at 20 km scale. This is due to its deterministic nature, although it is designed to be a nonlinear physical evolution scheme that is more powerful than the linear PySTEPS. We further boost the deterministic

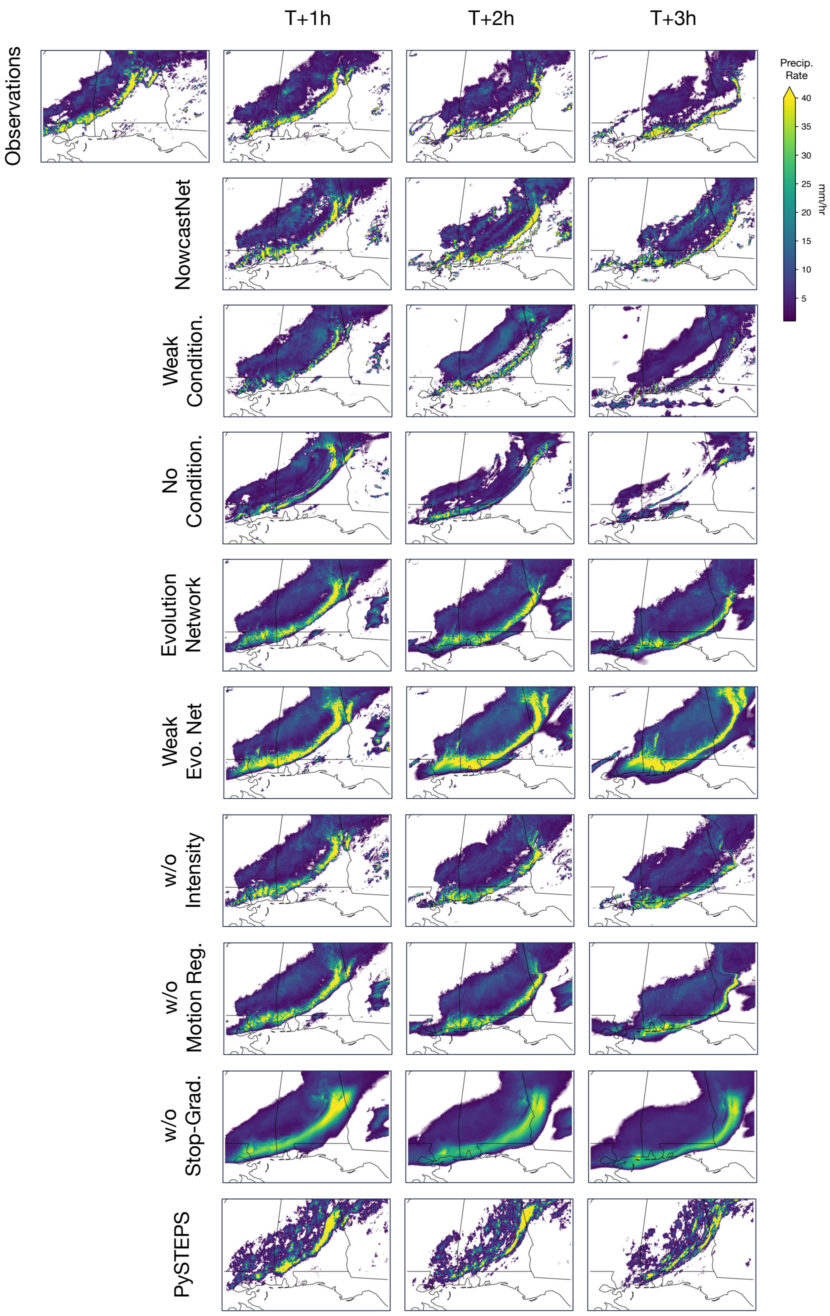

Supplementary Fig. 1: **Ablation study of NowcastNet on a precipitation event with squall line in eastern USA.** This case was studied comparatively in Extended Data Fig. 3. In reference to real observations, we inspect predictions of nine models: NowcastNet (Fig. 1a), NowcastNet w/ weak conditioning, NowcastNet w/o conditioning; Evolution Network (Fig. 1b), Evolution Network w/o accumulation loss, Evolution Network w/o intensity component, Evolution Network w/o motion regularization term, Evolution Network w/o stop-gradient; PySTEPS. Precipitation data obtained from MRMS [1] and maps produced with Cartopy and Natural Earth.

evolution network by the stochastic generative network, which makes the final NowcastNet able to generate convective details at 1-2 km scale.

We first consider a weaker version of the evolution network that does not adopt the accumulation loss. Instead, the weaker evolution network tries to predict the ground truth motion fields and intensity residues, by using as its objective the distance between ground truth $\widehat{\mathbf{x}}_{t+1}$ and the result of evolution on ground truth $\widehat{\mathbf{x}}_t$, which is not available at inference. Note that in our final accumulation loss, we adopt the distance between ground truth $\widehat{\mathbf{x}}_{t+1}$ and predicted field $\widehat{\mathbf{x}}''_{t+1}$, the result of evolution on previous prediction $\widehat{\mathbf{x}}''_t$, which exactly complies with the autoregressive nature of the physical evolution process. The other training settings are exactly the same as the original evolution network.

As shown in Supplementary Fig. 1, the weaker evolution network (6th row) produces predictions with serious and unacceptable location bias. We also find that the location bias has moderate similarity with the PySTEPS predictions (10th row). In particular, the locations of the head and tail of the squall line predicted by the weaker evolution network and PySTEPS are fairly consistent. This is a strong evidence that PySTEPS lacks an end-to-end error optimization framework to explicitly control the accumulated error in the autoregressive evolution process.

In contrast, the original evolution network (5th row) yields significantly better predictions of the motion and the shape of the squall line. The high nowcast skill stems from its capability of integrating the physical evolution scheme into a differentiable neural evolution operator (Fig. 1c) and empowering a fully neural framework with nonlinear simulations and end-to-end error optimization (Fig. 1b).

Second, we consider another weaker version of the evolution network by dropping the intensity-residual component. As shown in Supplementary Fig. 1, the motion-only evolution network (7th row) is better able to provide plausible location predictions consistent with the ground truth. This confirms the evolution network, benefiting from its particular designs (Fig. 1b), achieves essential improvements over PySTEPS.

Third, we consider another weaker version of the evolution network by dropping the motion regularization term (8th row). This term is defined by an assumption in the motion field estimation that all neighbor points have similar motions, which is motivated in part by the continuity equation and in part by the fact that heavy precipitation patterns tend to be longer-lived and stabler in movement [2]. As shown in Supplementary Fig. 1 (8th row), the squall line predicted by this weaker version becomes unnaturally distorted after two hours. Note that the motion regularization term cannot be used independently because the accumulation loss to control the forecast error is the pillar objective of our physical evolution scheme.

Finally, we inspect the last weaker version of the evolution network by eliminating the stop-gradient mechanism in the neural evolution operator (Fig. 1c), which is originally designed to mitigate the numerical instability.

As shown in Supplementary Fig. 1, the weaker network without stopping the gradients (9th row) incurs serious location bias and unnatural dissipation. This verifies the need of the stop-gradient design for the neural evolution operator, which underlies the stable learnability of NowcastNet.

B Evaluation metrics

We first describe the four standard evaluation metrics used in this article.

Critical Success Index (CSI) [3] measures the accuracy of the binary decision induced by the predicted field whether the rain rate exceeds a particular threshold τ. It is computed by the ratio of the hit on the rain rate exceeding the threshold in the sum of hit, miss and false alarm on all grids [4]. It considers the precision and the recall simultaneously, and is a common choice for evaluation in precipitation nowcasting. It is noteworthy that during computing the CSI metric, the hit, miss and false alarm are counted over all the test set.

CSI-Neighborhood [4] is the CSI metric calculated based on the neighborhood. The neighborhood methods evaluate the forecasts within a spatial window surrounding each grid, and can enable verification of how "close" the forecasts are, which are particularly suited for verifying high-resolution forecasts. CSI-Neighborhood calculates the counts of hit, miss and false alarm after max pooling with kernel size κ and stride $\lfloor \kappa/2 \rfloor$.

Fractional Skill Score (FSS) [5] is another metric based on the neighborhood, defined by a rain rate threshold τ. For every grid in the test dataset, we compute the fraction of surrounding grids within a spatial window that have exceeded the threshold. Then we sum the difference between the fractions of predictions and observations for all grids in the dataset, referred to as Fractional Brief Score (FBS). FSS is calculated from the normalized FBS, relying on τ to decide whether it rains on a local grid and use the Fractions Brier Score (FBS) to compare the forecasted and observed rain frequencies. Compared to CSI-Neighborhood that focuses on whether the prediction makes successful hits, FSS can further compare the ratio of grids that exceed the threshold in the spatial windows.

Power Spectral Density (PSD) [6, 7] measures the power distribution over each spatial frequency, comparing the precipitation variability of forecasts to that of the observations. We adopt the implementation of PSD from the PySTEPS package [8]. Forecasts that have more minor differences with observations are less preferred.

C Additional precipitation events

We study additional cases covering both extreme and ordinary precipitation events in the USA and China, which are selected from the test set with the help of five chief forecasters at the China Meteorological Administration to be representative events in the periods for evaluation.

Selected precipitation events of 2021 in the USA are shown in Extended Data Fig. 2–6 and Supplementary Fig. 2–5, including the event with tornado on March 25 [1], the event with a massive squall line on May 4 [2], the event with convective initiation and dissipation on August 14, the remnants of Hurricane Ida on September 1 [3], the event with tornado outbreak on December 11 [4], the event with widespread precipitation on April 11, the event with multiple supercells on April 24 [5], the event with isolated cells on June 12, and the event with a linear storm system on July 14 [6]. Selected precipitation events from April to June 2021 in China are shown in Extended Data Fig. 7–8, including the hail event caused by a squall line on May 3 and the gale-warning event along with hail and lightning on June 30.

We further demonstrate in Extended Data Fig. 9–10 the high-resolution precipitation nowcasts over a larger spatial region of 2048 km $\times$ 2048 km, corresponding to the USA and China precipitation events in Fig. 2–3 of the main text, respectively.

Videos of the selected precipitation events in the main text, the Extended Data and the Supplementary Information, and the corresponding three-hour nowcasts of different models are given in Supplementary Movie 1–13.

Showcases of the randomly-sampled events used in the meteorologist evaluation with the expert choices are shown in Supplementary Fig. 6–7 for the USA events and in Supplementary Fig. 8–9 for the China events.

[1] https://www.weather.gov/ohx/20210325
[2] https://www.weather.gov/bmx/event_05042021
[3] https://www.weather.gov/phi/eventreview20210901
[4] https://www.weather.gov/pah/December-10th-11th-2021-Tornado
[5] https://www.weather.gov/tae/20210424_tornadoes
[6] https://www.weather.gov/arx/jul1421

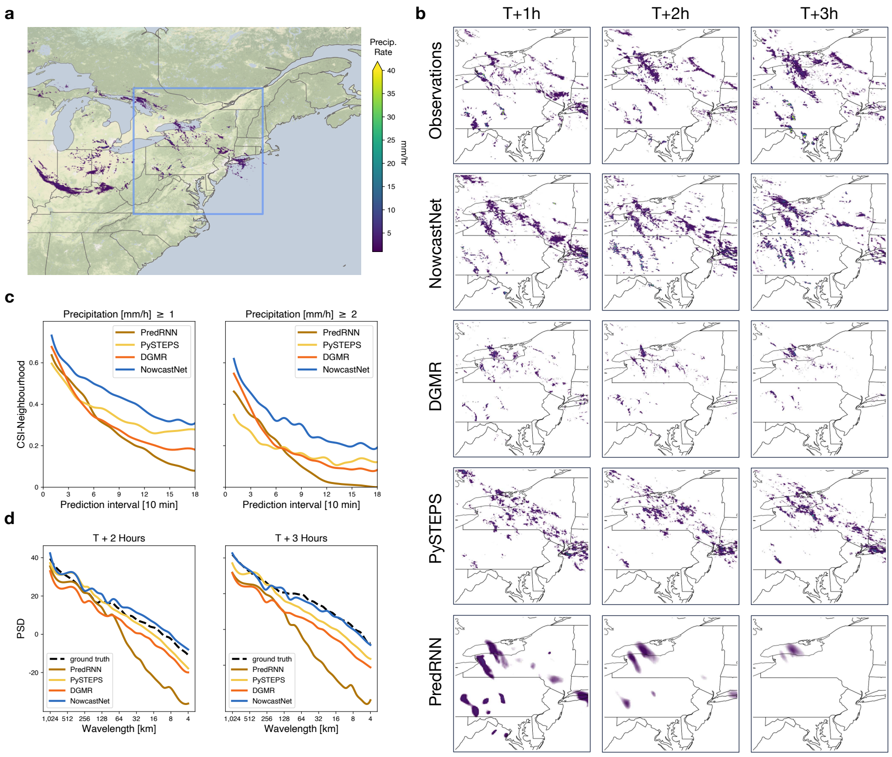

Supplementary Fig. 2: **Case study of a moderate precipitation event starting on Apr 11th 20:50 UTC, 2021, with widespread, scattered rainfall sustained for several hours around the eastern USA.** NowcastNet can predict the scattered locations of precipitation with better coverage and details. **a**, Geographic context for the predictions. **b**, A single prediction at $T+1$, $T+2$ and $T+3$ hour lead times for different models. **c**, CSI-neighborhood at thresholds 1 mm/h and 2 mm/h. **d**, PSD at different wavelengths. Images are zoomed in 768 km $\times$ 768 km to highlight local details. Precipitation data obtained from MRMS [1] and maps produced with Cartopy and Natural Earth.

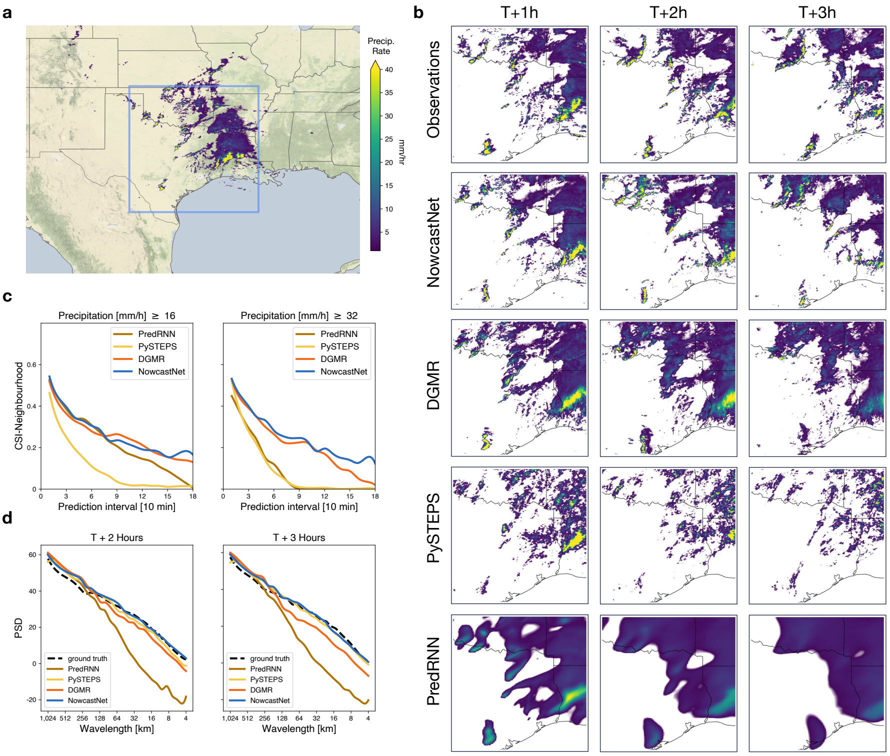

Supplementary Fig. 3: **Case study of an extreme precipitation event starting on Apr 24th 00:30 UTC, 2021, with multiple supercells converging to squall lines around the east of Texas.** NowcastNet is the only model providing accurate nowcasts of both location and intensity for the evolution of the multiple supercells. **a**, Geographic context for the predictions. **b**, A single prediction at $T+1$, $T+2$ and $T+3$ hour lead times for different models. **c**, CSI-neighborhood at thresholds 16 mm/h and 32 mm/h. **d**, PSD at different wavelengths. Images are zoomed in 768 km $\times$ 768 km to highlight local details. Precipitation data obtained from MRMS [1] and maps produced with Cartopy and Natural Earth.

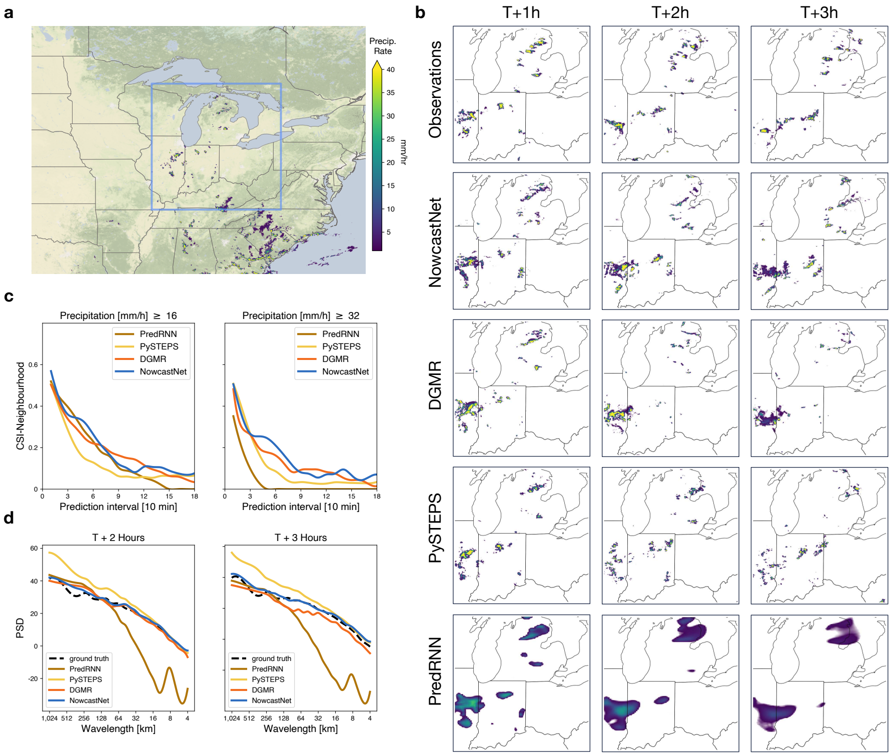

Supplementary Fig. 4: **Case study of a moderate precipitation event starting on Jun 12th 22:20 UTC, 2021, with isolated cells affecting Illinois, Michigan, and Indiana.** NowcastNet avoids undesired dissipation and provides more plausible predictions for the range and location of the isolated cells. **a**, Geographic context for the predictions. **b**, A single prediction at $T+1$, $T+2$ and $T+3$ hour lead times for different models. **c**, CSI-neighborhood at thresholds 16 mm/h and 32 mm/h. **d**, PSD at different wavelengths. Images are zoomed in 768 km × 768 km to highlight local details. Precipitation data obtained from MRMS [1] and maps produced with Cartopy and Natural Earth.

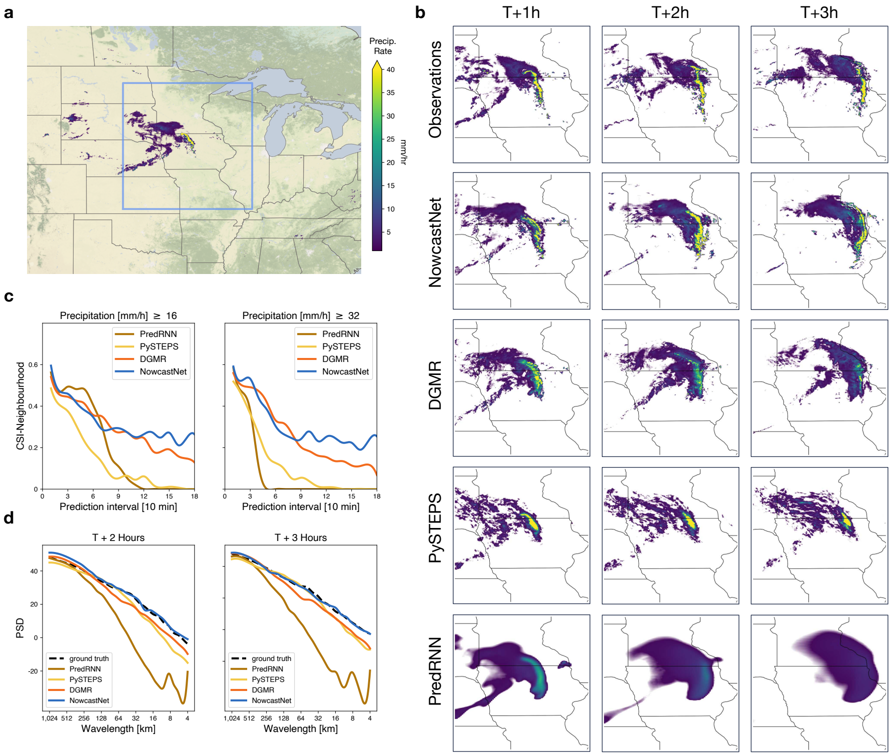

Supplementary Fig. 5: Case study of an extreme precipitation event starting on July 14th 13:30 UTC, 2021, with a line of thunderstorms moving across Wisconsin causing strong winds and heavy rainfalls. NowcastNet gives better and stabler nowcasts on the rotation and intensity of the line echo. **a**, Geographic context for the predictions. **b**, A single prediction at $T + 1$, $T + 2$ and $T + 3$ hour lead times for different models. **c**, CSI-neighborhood at thresholds 16 mm/h and 32 mm/h. **d**, PSD at different wavelengths. Images are zoomed in 768 km $\times$ 768 km to highlight local details. Precipitation data obtained from MRMS [1] and maps produced with Cartopy and Natural Earth.

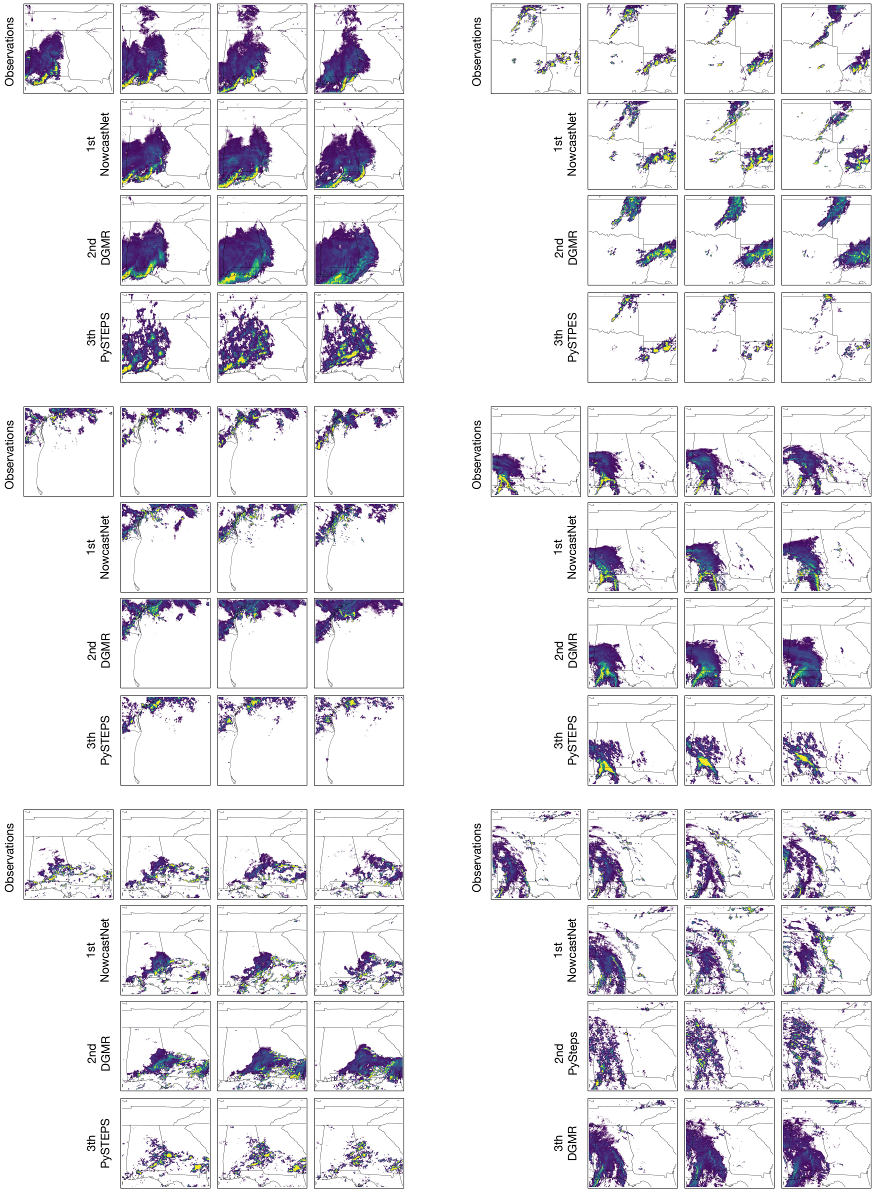

Supplementary Fig. 6: **Meteorologist evaluation on precipitation events randomly sampled from the USA dataset.** Each subfigure shows an event. Nowcasts by PredRNN-V2, PySTEPS, DGMR, and NowcastNet are ranked by expert meteorologists and the top three places are listed. Precipitation data obtained from MRMS [1] and maps produced with Cartopy and Natural Earth.

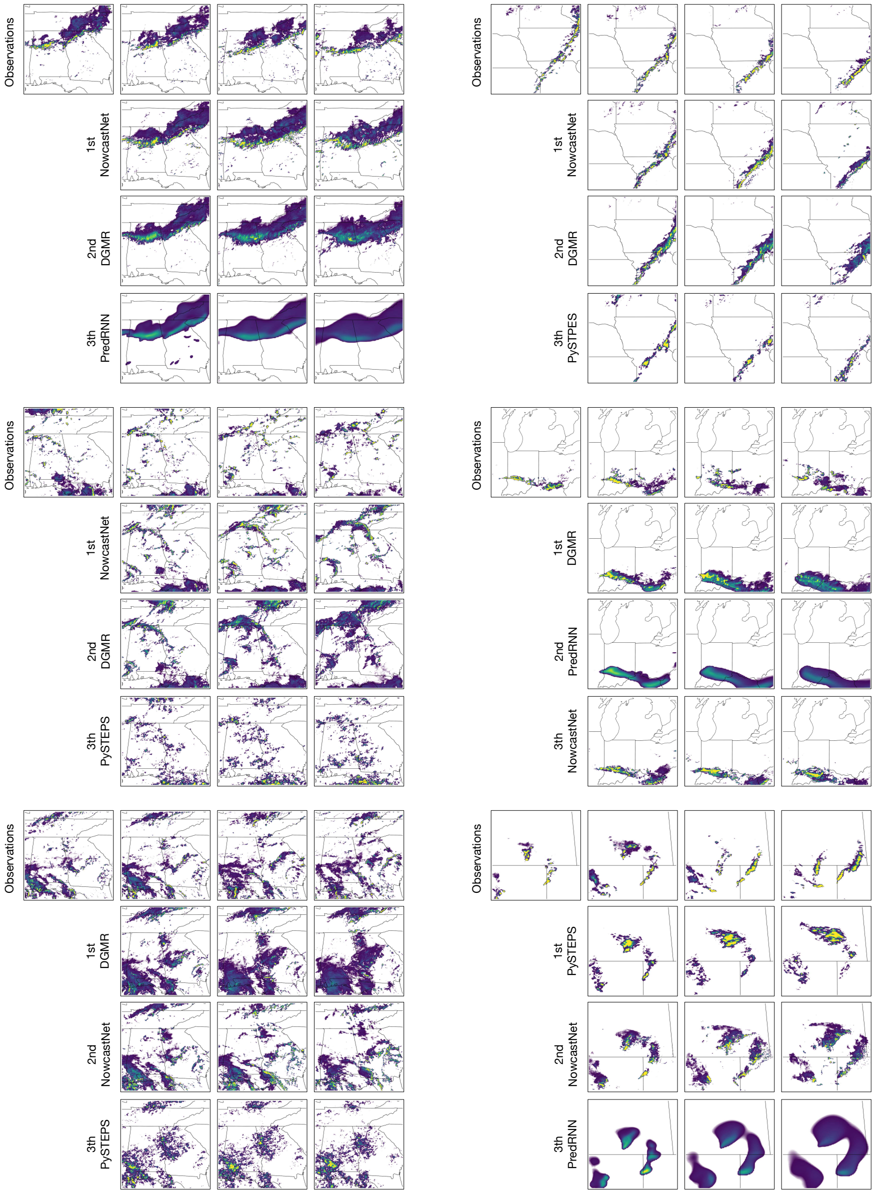

Supplementary Fig. 7: **Meteorologist evaluation on precipitation events randomly sampled from the USA dataset.** Each subfigure shows an event. Nowcasts by PredRNN-V2, PySTEPS, DGMR, and NowcastNet are ranked by expert meteorologists and the top three places are listed. Precipitation data obtained from MRMS [1] and maps produced with Cartopy and Natural Earth.

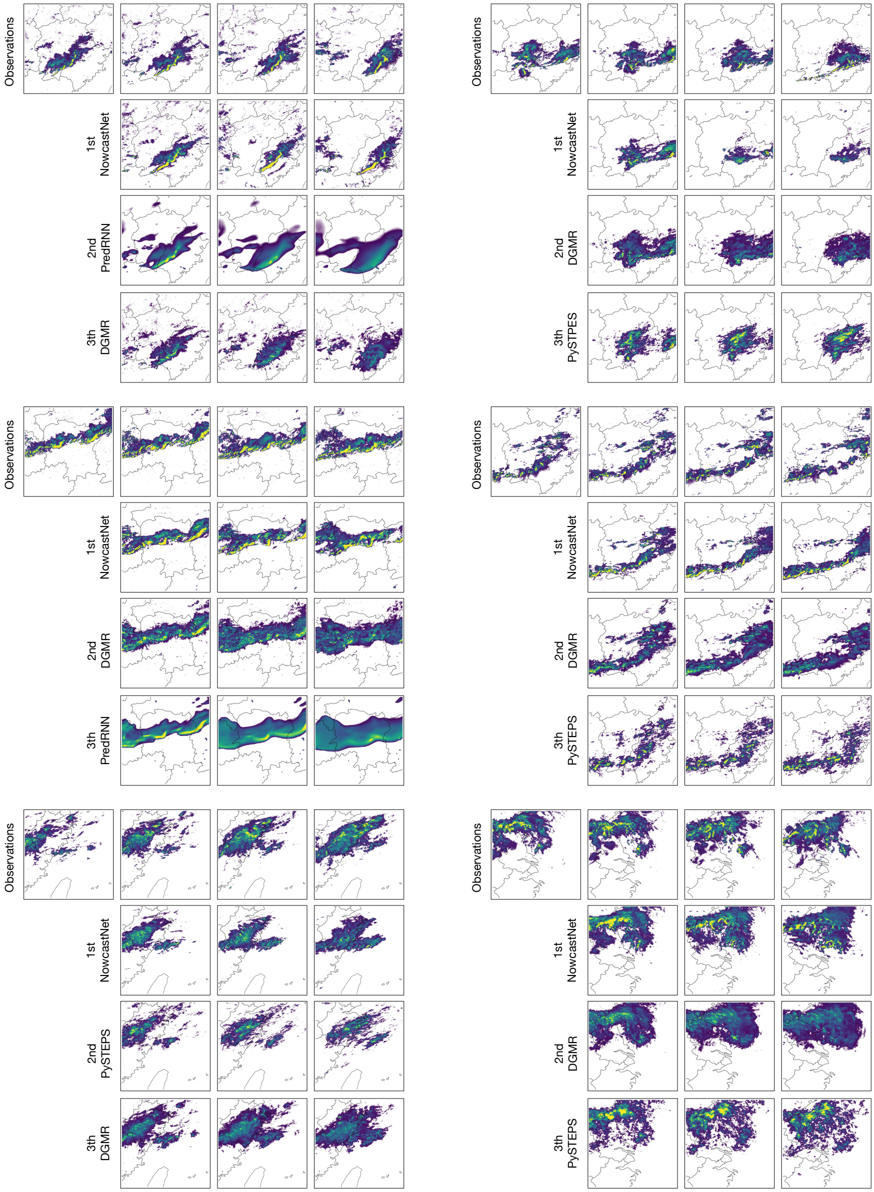

Supplementary Fig. 8: **Meteorologist evaluation on precipitation events randomly sampled from the China dataset.** Each subplot shows an event. Nowcasts by PredRNN-V2, PySTEPS, DGMR, and NowcastNet are ranked by expert meteorologists and the top three places are listed. Precipitation data is obtained from China dataset and maps produced with Cartopy and Natural Earth.

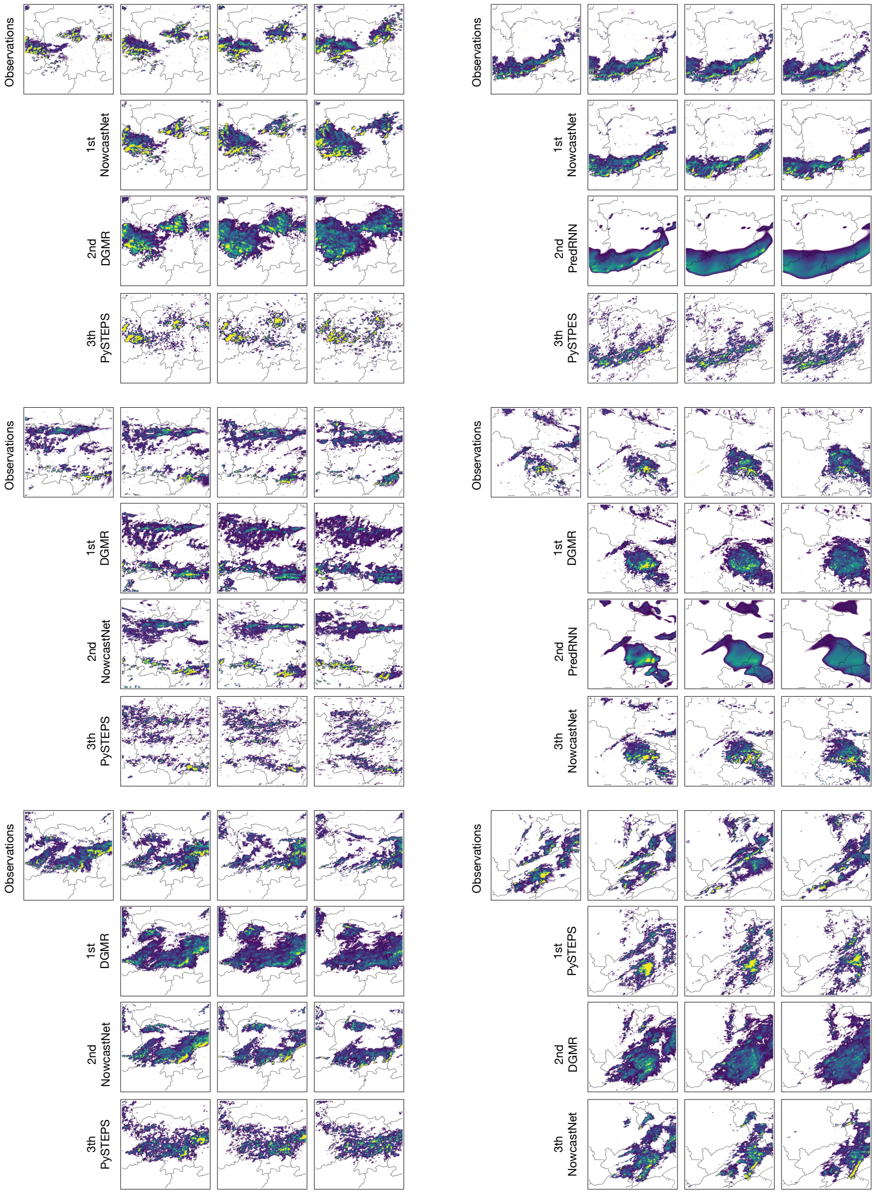

Supplementary Fig. 9: **Meteorologist evaluation on precipitation events randomly sampled from the China dataset.** Each subplot shows an event. Nowcasts by PredRNN-V2, PySTEPS, DGMR, and NowcastNet are ranked by expert meteorologists and the top three places are listed. Precipitation data is obtained from China dataset and maps produced with Cartopy and Natural Earth.

D Additional quantitative results

We then present additional quantitative results on the uniform-sampled test set in Supplementary Fig. 10–11, in which we include results of the evolution network termed as NowcastNet-evo. In addition to the quantitative results shown in the main text, we present additional results in the other metrics on the importance-sampled USA test set in Supplementary Fig. 12–14, and that on the importance-sampled China test set in Supplementary Fig. 15–17.

We only present the results of NowcastNet with the strongest baselines, DGMR and PySTEPS. NowcastNet is comparable to DGMR at the rain rates below 8 mm/h but is preferable at higher rain rates. The quantitative results verify that NowcastNet is generally useful for light-to-heavy precipitation and particularly skillful for extreme precipitation. As the neighborhood size in these metrics gets larger, i.e., higher focus on the physical plausibility [9], the advantage of NowcastNet becomes more prominent.

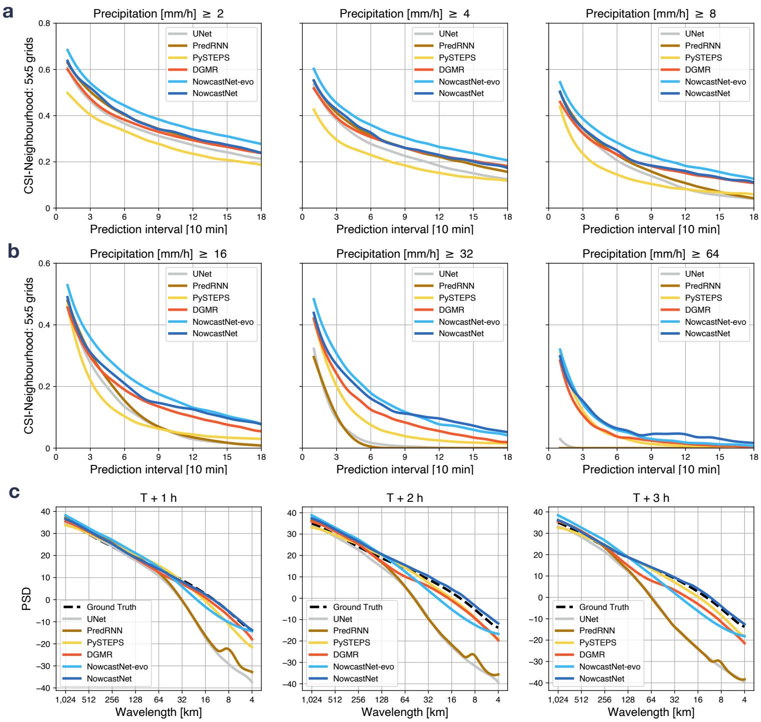

Supplementary Fig. 10: **Verification scores for the USA events in 2021 under the uniform-sampling protocol. a**, CSI-neighborhood of different models at precipitation thresholds of 2 mm/h, 4 mm/h, and 8 mm/h. **b**, CSI-neighborhood of different models at precipitation thresholds of 16 mm/h, 32 mm/h, and 64 mm/h. **c**, Radially-averaged PSD over 1,024 km $\times$ 1,024 km predictions for all models at T+1 hour, T+2 hour, and T+3 hour.

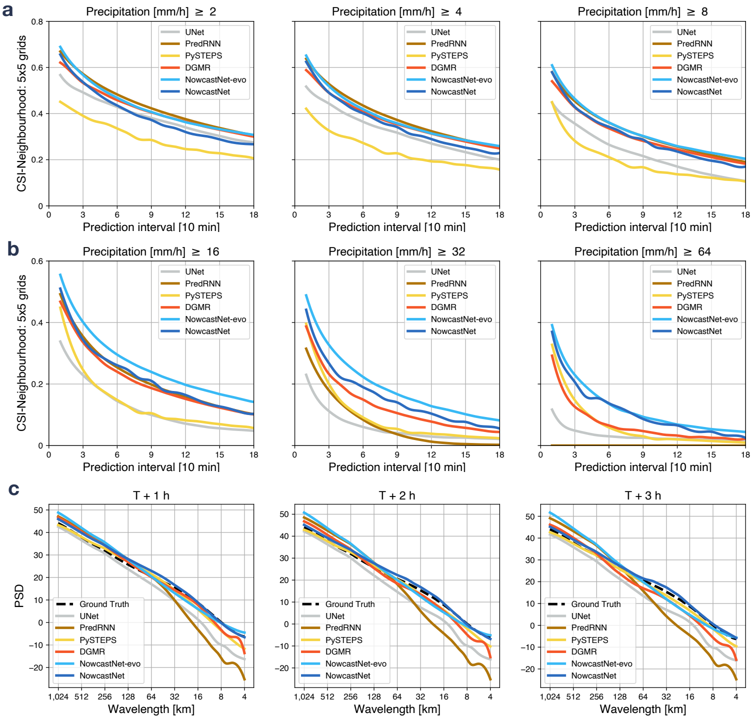

Supplementary Fig. 11: **Verification scores for the China events in 2021 under the uniform-sampling protocol. a**, CSI-neighborhood of different models at precipitation thresholds of 2 mm/h, 4 mm/h, and 8 mm/h. **b**, CSI-neighborhood of different models at precipitation thresholds of 16 mm/h, 32 mm/h, and 64 mm/h. **c**, Radially-averaged PSD over 1,024 km $\times$ 1,024 km predictions for all models at T+1 hour, T+2 hour, and T+3 hour.

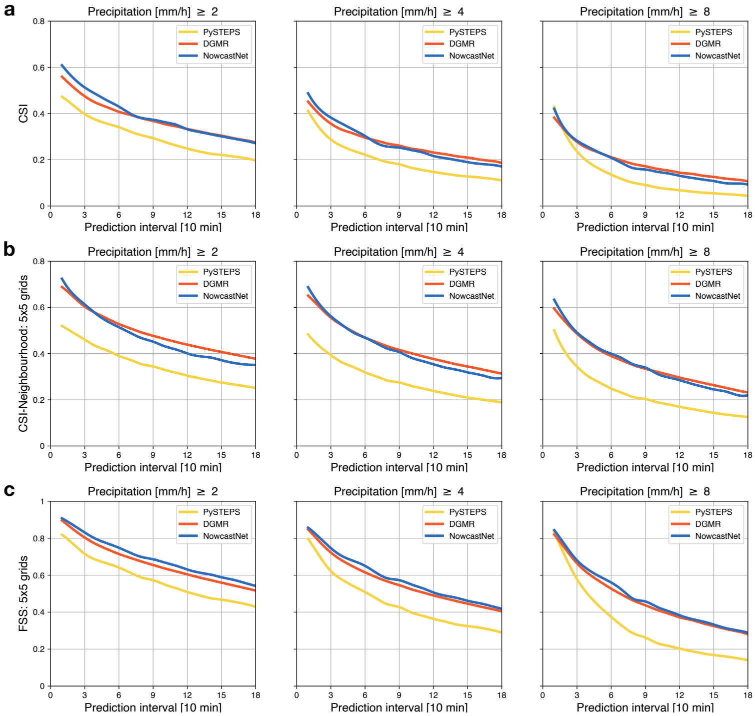

Supplementary Fig. 12: **Verification scores for the USA dataset under the importance-sampling protocol**. **a**, CSI of different models with precipitation thresholds 2 mm/h, 4 mm/h, 8 mm/h. **b**, CSI-Neighborhood of different models with precipitation thresholds 2 mm/h, 4 mm/h, 8 mm/h, and average rain rate over a neighborhood of 5×5 grids. **c**, FSS of different models with precipitation thresholds 2 mm/h, 4 mm/h, 8 mm/h, and average rain rate over a neighborhood of 5×5 grids.

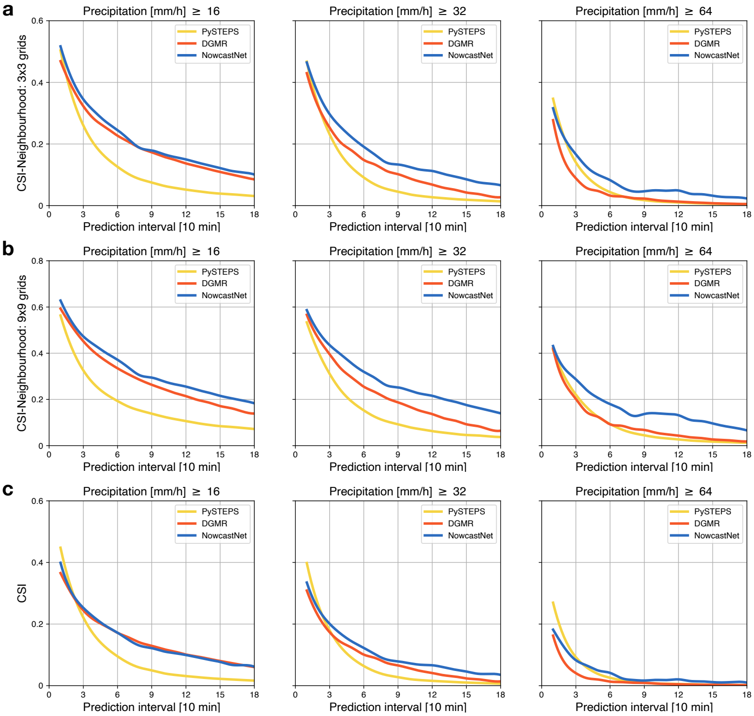

Supplementary Fig. 13: **Verification scores for the USA dataset under the importance-sampling protocol. a**, CSI-Neighborhood of different models with precipitation thresholds 16 mm/h, 32 mm/h, 64 mm/h, and average rain rate over a neighborhood of 3×3 grids. **b**, CSI-Neighborhood of different models with precipitation thresholds 16 mm/h, 32 mm/h, 64 mm/h, and average rain rate over a neighborhood of 9×9 grids. **c**, FSS of different models with precipitation thresholds 2 mm/h, 4 mm/h, 8 mm/h, and average rain rate over a neighborhood of 5×5 grids.

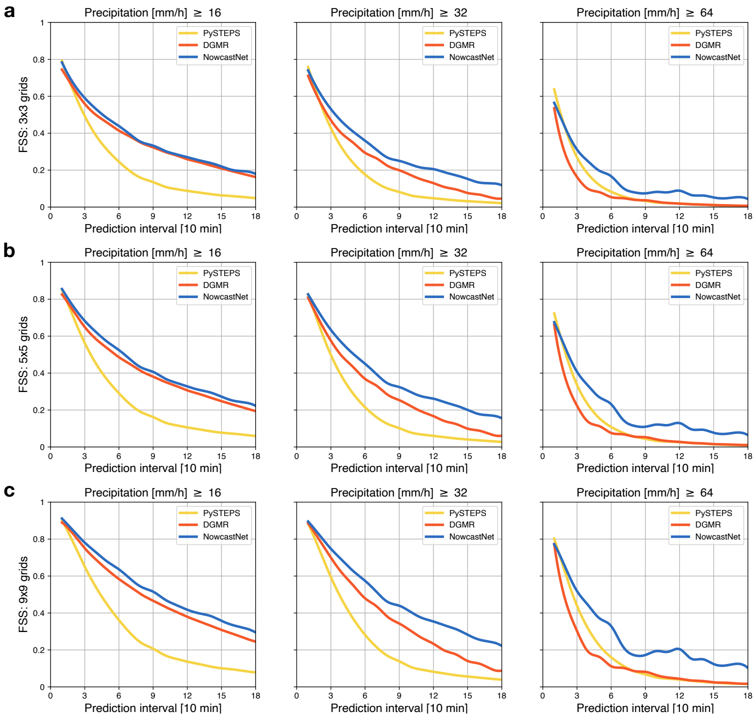

Supplementary Fig. 14: **Verification scores for the USA dataset under the importance-sampling protocol.** FSS of different models with precipitation thresholds 16 mm/h, 32 mm/h, 64 mm/h, taking average rain rate over a neighborhood of 3×3 grids in **a**, 5×5 grids in **b**, and 9×9 grids in **c**.

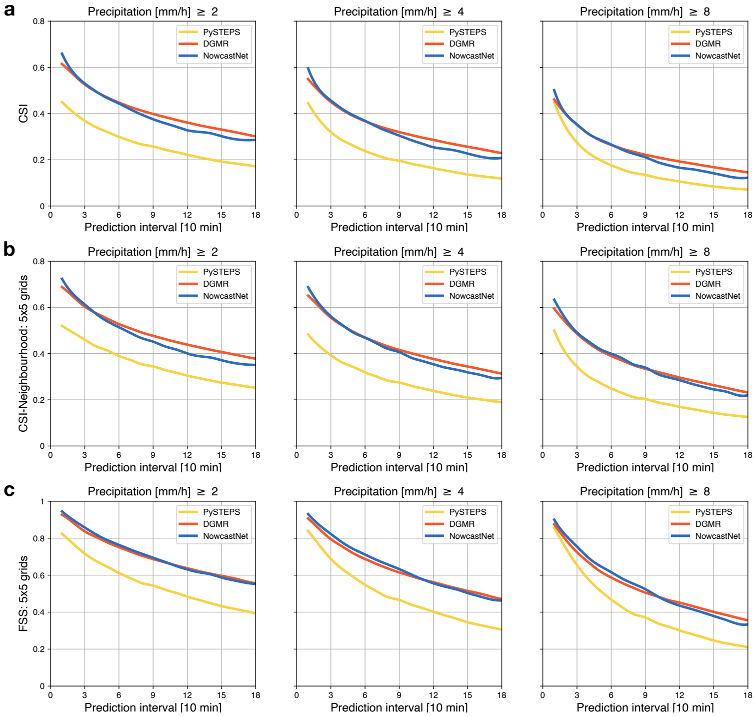

Supplementary Fig. 15: **Verification scores for the China dataset under the importance-sampling protocol. a**, CSI of different models with precipitation thresholds 2 mm/h, 4 mm/h, 8 mm/h. **b**, CSI-Neighborhood of different models with precipitation thresholds 2 mm/h, 4 mm/h, 8 mm/h, and average rain rate over a neighborhood of 5×5 grids. **c**, FSS of different models with precipitation thresholds 2 mm/h, 4 mm/h, 8 mm/h, and average rain rate over a neighborhood of 5×5 grids.

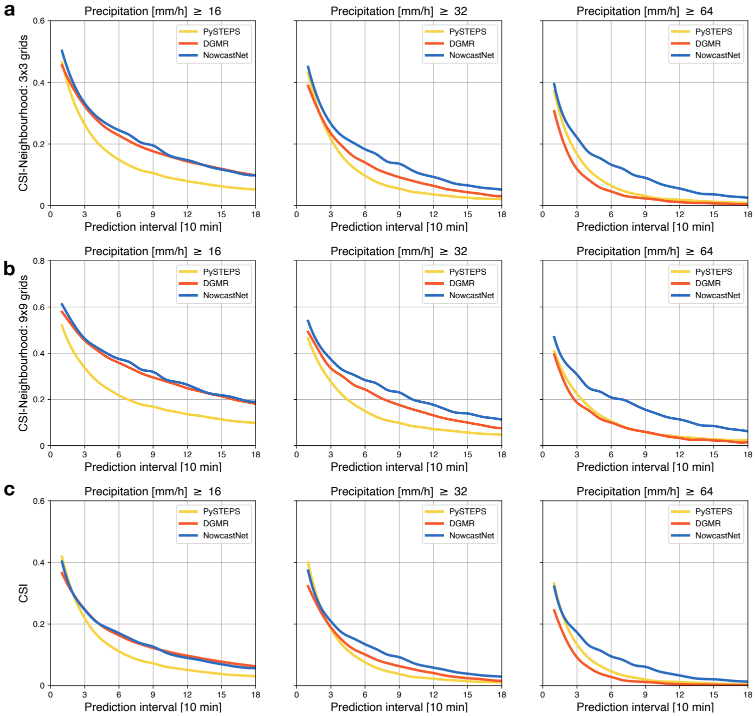

Supplementary Fig. 16: **Verification scores for the China dataset under the importance-sampling protocol. a**, CSI-Neighborhood of different models with precipitation thresholds 16 mm/h, 32 mm/h, 64 mm/h, and average rain rate over a neighborhood of 3 × 3 grids. **b**, CSI-Neighborhood of different models with precipitation thresholds 16 mm/h, 32 mm/h, 64 mm/h, and average rain rate over a neighborhood of 9 × 9 grids. **c**, FSS of different models with precipitation thresholds 2 mm/h, 4 mm/h, 8 mm/h, and average rain rate over a neighborhood of 5 × 5 grids.

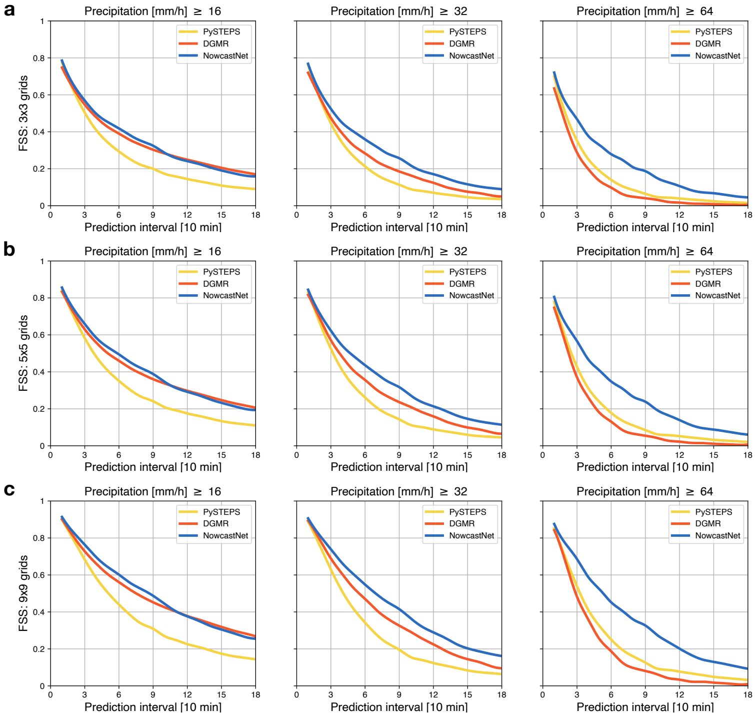

Supplementary Fig. 17: **Verification scores for the China dataset under the importance-sampling protocol.** FSS of different models with precipitation thresholds 16 mm/h, 32 mm/h, 64 mm/h, taking average rain rate over a neighborhood of 3×3 grids in **a**, 5×5 grids in **b**, and 9×9 grids in **c**.

E Related work

We elaborate a more extensive review on the development of precipitation nowcasting methods, and highlight their differences to NowcastNet.

In advection scheme-based methods, Lagrangian persistence is a basic paradigm [10], where the current state of the motion field is first estimated by optical flow methods and then used to advect the current state of radar reflectivity field for future time steps. Since the spatial scale of the precipitation patterns largely determines the temporal duration of its persistence [10], multiscale methods such as S-PROG [11] with Fourier decomposition or MAPLE [12] with wavelet decomposition are further proposed to predict the stochastic intensity evolution in the advection process. Another line of work considers the spectral method that solves for the motion and intensity fields from the continuity equation by Fourier transform [13, 14]. STEPS [15, 16] models both the stochastic intensity evolution and the stochastic perturbation of the motion fields, which is widely deployed as a strong precipitation nowcasting model. Many operational methods of the advection scheme are implemented in the popular open-source packages such as PySTEPS [8] or Rainymotion [17].

The first deep architecture designed for precipitation nowcasting is Convolutional LSTM [18], which unifies convolutional and recurrent structures for spatiotemporal prediction. This model was extensively improved later from multiple aspects, such as introducing the predictive coding structure [19], adding spatial-temporal highway [20, 21] or involving learnable feature deformations in the prediction process [22, 23]. These methods are used as common baselines for data-driven precipitation nowcasting [24–26]. U-Net [27], a general multiscale backbone widely used in Artificial Intelligence for Science (AI4Science), is modified for precipitation nowcasting [28, 29]. Another series of methods, including MetNet and MetNet-V2 [30, 31], have further provided rainfall predictions of larger spatial range and longer lead times but the crucial multiscale patterns useful for meteorologists are lost significantly.

Previous methods suffer inevitably from unnatural blur and dissipation in the predictions that lose most of convective-scale features. To mitigate this problem, a special loss from the computer vision area is adopted in [32] to enhance the field sharpness. Adversarial learning techniques are also involved to tackle the problem of blurry predictions [33, 34]. On extreme precipitation events, the stacked neural networks on different rain levels are used in [35], which can reach the same forecasting skill of advection scheme method S-PROG [11] on heavy precipitation in one-hour lead time. DGMR [9], a major step forward by DeepMind, explores generative models in precipitation nowcasting by integrating spatiotemporal consistency and log-normal distribution of rain rate, and enables probabilistic predictions and ensemble forecasting. Since DGMR is proved to be the state-of-the-art model in the expert examination held by the UK Met-Office, we consider it as a powerful competing method in this article.

Deep learning models inspired by physical principles are another class of related work. Physics-informed neural networks (PINN) [36] are created based on partial differential equations (PDE). PINN leverages the gradient-solving ability of neural networks to approximate the differential equation by taking it as the objective function. Neural ordinary differential equation (NODE) [37] adopts the numerical methods such as Runge-Kutta to replace the forward propagation in neural networks. NODE is a powerful parameterization tool for modeling continuous processes. The idea of integrating physical knowledge into deep learning was studied for the predictions of sea surface temperature fields [38]. Here, the method uses a deep model to predict motion field for a single time step forward from the past observations, and the temperature field is advected by the motion field; Such a process is repeated autoregressively. This method relies on the autoregressive solving process of the equation, incurring uncontrollable accumulation error. The method has not been shown skillful for extreme precipitation nowcasting.

References

[1] Zhang, J. *et al.* Multi-radar multi-sensor (MRMS) quantitative precipitation estimation: Initial operating capabilities. *Bulletin of the American Meteorological Society* **97** (4), 621–638 (2016).

[2] Pierce, C., Seed, A., Ballard, S., Simonin, D. & Li, Z. in *Nowcasting* (eds Bech, J. & Chau, J. L.) *Doppler Radar Observations* Ch. 4 (IntechOpen, Rijeka, 2012).

[3] Schaefer, J. T. The critical success index as an indicator of warning skill. *Weather and Forecasting* **5** (4), 570–575 (1990).

[4] Jolliffe, I. T. & Stephenson, D. B. *Forecast Verification: A Practitioner's Guide in Atmospheric Science* (John Wiley & Sons, 2012).

[5] Roberts, N. M. & Lean, H. W. Scale-selective verification of rainfall accumulations from high-resolution forecasts of convective events. *Monthly Weather Review* **136** (1), 78–97 (2008).

[6] Harris, D., Foufoula-Georgiou, E., Droegemeier, K. K. & Levit, J. J. Multiscale statistical properties of a high-resolution precipitation forecast. *Journal of Hydrometeorology* **2** (4), 406–418 (2001).

[7] Sinclair, S. & Pegram, G. Empirical mode decomposition in 2-d space and time: a tool for space-time rainfall analysis and nowcasting. *Hydrology and Earth System Sciences* **9** (3), 127–137 (2005).

[8] Pulkkinen, S. *et al.* Pysteps: an open-source python library for probabilistic precipitation nowcasting (v1.0). *Geoscientific Model Development* **12** (10), 4185–4219 (2019).

[9] Ravuri, S. *et al.* Skilful precipitation nowcasting using deep generative models of radar. *Nature* **597** (7878), 672–677 (2021).

[10] Germann, U. & Zawadzki, I. Scale-dependence of the predictability of precipitation from continental radar images. Part I: Description of the methodology. *Monthly Weather Review* **130** (12), 2859–2873 (2002).

[11] Seed, A. A dynamic and spatial scaling approach to advection forecasting. *Journal of Applied Meteorology* **42** (3), 381–388 (2003).

[12] Turner, B., Zawadzki, I. & Germann, U. Predictability of precipitation from continental radar images. part iii: Operational nowcasting implementation (maple). *Journal of Applied Meteorology and Climatology* **43** (2), 231–248 (2004).

[13] Xu, G. & Chandrasekar, V. Radar storm motion estimation and beyond: A spectral algorithm and radar observation based dynamic model (2005).

[14] Ruzanski, E., Chandrasekar, V. & Wang, Y. The casa nowcasting system. *Journal of Atmospheric and Oceanic Technology* **28** (5), 640–655 (2011).

[15] Bowler, N. E., Pierce, C. E. & Seed, A. W. Steps: A probabilistic precipitation forecasting scheme which merges an extrapolation nowcast with downscaled nwp. *Quarterly Journal of the Royal Meteorological Society* **132** (620), 2127–2155 (2006).

[16] Seed, A. W., Pierce, C. E. & Norman, K. Formulation and evaluation of a scale decomposition-based stochastic precipitation nowcast scheme. *Water Resources Research* **49** (10), 6624–6641 (2013).

[17] Ayzel, G., Heistermann, M. & Winterrath, T. Optical flow models as an open benchmark for radar-based precipitation nowcasting (rainymotion v0.1). *Geoscientific Model Development* **12** (4), 1387–1402 (2019).

[18] Shi, X. *et al.* Convolutional LSTM network: A machine learning approach for precipitation nowcasting. *Advances in Neural Information Processing Systems* **28** (2015).

[19] Lotter, W., Kreiman, G. & Cox, D. A neural network trained for prediction mimics diverse features of biological neurons and perception. *Nature Machine Intelligence* **2** (4), 210–219 (2020).

[20] Wang, Y. *et al.* PredRNN: A recurrent neural network for spatiotemporal predictive learning. *IEEE Transactions on Pattern Analysis and Machine Intelligence* **45** (2), 2208–2225 (2022).

[21] Wang, Y., Gao, Z., Long, M., Wang, J. & Yu, P. S. PredRNN++: Towards a resolution of the deep-in-time dilemma in spatiotemporal predictive learning. *International Conference on Machine Learning* **80**, 5123–5132 (2018).

[22] Shi, X. *et al.* Deep learning for precipitation nowcasting: A benchmark and a new model. *Advances in Neural Information Processing Systems* **30** (2017).

[23] Wu, H., Yao, Z., Wang, J. & Long, M. MotionRNN: A flexible model for video prediction with spacetime-varying motions. *IEEE Conference on Computer Vision and Pattern Recognition* 15435–15444 (2021).

[24] Bonnet, S. M., Evsukoff, A. & Morales Rodriguez, C. A. Precipitation nowcasting with weather radar images and deep learning in são paulo, brasil. *Atmosphere* **11** (11), 1157 (2020).

[25] Marrocu, M. & Massidda, L. Performance comparison between deep learning and optical flow-based techniques for nowcast precipitation from radar images. *Forecasting* **2** (2), 194–210 (2020).

[26] Franch, G. *et al.* Taasrad19, a high-resolution weather radar reflectivity dataset for precipitation nowcasting. *Scientific Data* **7** (1), 1–13 (2020).

[27] Ronneberger, O., Fischer, P. & Brox, T. U-net: Convolutional networks for biomedical image segmentation. *International Conference on Medical Image Computing and Computer-Assisted Intervention* 234–241 (2015).

[28] Agrawal, S. *et al.* Machine learning for precipitation nowcasting from radar images. *arXiv preprint arXiv:1912.12132* (2019).

[29] Ayzel, G., Scheffer, T. & Heistermann, M. Rainnet v1.0: a convolutional neural network for radar-based precipitation nowcasting. *Geoscientific Model Development* **13** (6), 2631–2644 (2020).

[30] Sønderby, C. K. *et al.* Metnet: A neural weather model for precipitation forecasting. *arXiv preprint arXiv:2003.12140* (2020).

[31] Espeholt, L. *et al.* Skillful twelve hour precipitation forecasts using large context neural networks. *arXiv preprint arXiv:2111.07470* (2021).

[32] Tran, Q.-K. & Song, S.-K. Computer vision in precipitation nowcasting: Applying image quality assessment metrics for training deep neural networks. *Atmosphere* **10** (5), 244 (2019).

[33] Jing, J., Li, Q. & Peng, X. MLC-LSTM: Exploiting the spatiotemporal correlation between multi-level weather radar echoes for echo sequence extrapolation. *Sensors* **19** (18), 3988 (2019).

[34] Liu, H.-B. & Lee, I. Mpl-gan: Toward realistic meteorological predictive learning using conditional gan. *IEEE Access* **8**, 93179–93186 (2020).

[35] Franch, G. *et al.* Precipitation nowcasting with orographic enhanced stacked generalization: Improving deep learning predictions on extreme events. *Atmosphere* **11** (3), 267 (2020).

[36] Raissi, M., Perdikaris, P. & Karniadakis, G. E. Physics-informed neural networks: A deep learning framework for solving forward and inverse problems involving nonlinear partial differential equations. *Journal of Computational Physics* **378**, 686–707 (2019).

[37] Chen, R. T. Q., Rubanova, Y., Bettencourt, J. & Duvenaud, D. K. Neural ordinary differential equations. *Advances in Neural Information Processing Systems* **31** (2018).

[38] de Bezenac, E., Pajot, A. & Gallinari, P. Deep learning for physical processes: Incorporating prior scientific knowledge. *International Conference on Learning Representations* (2018).
